# Efficacy of non-pharmacological interventions for sleep quality in Parkinson’s disease: a systematic review and network meta-analysis

**DOI:** 10.3389/fnins.2024.1337616

**Published:** 2024-02-21

**Authors:** Rongzhu Tang, Siyuan Gong, Jia Li, Wangjuan Hu, Jihong Liu, Chunlian Liao

**Affiliations:** Department of Neurology, The Second Affiliated Hospital of Chongqing Medical University, Chongqing, China

**Keywords:** non-pharmacological interventions, sleep quality, network meta-analysis, Parkinson’s disease, randomized controlled trials

## Abstract

**Background:**

Sleep disorders are one of the most common non-motor symptoms in PD. It can cause a notable decrease in quality of life and functioning in PD patients, as well as place a huge burden on both patients and caregivers. Currently, there are numerous non-pharmacological interventions available to improve sleep quality in PD, with disagreement as to which intervention is most effective. This network meta-analysis was performed to compare and rank non-pharmacological interventions to explore their efficacy in improving sleep quality in PD and to select the best interventions, with a view to providing references and bases for the development of clinical treatments and care programs.

**Methods:**

The PubMed, Embase, Cochrane Central Register of Controlled Trials (CENTRAL), Web of Science, China National Knowledge Infrastructure (CNKI), and Wanfang databases were searched from inception to December 6, 2023. Two authors independently screened all studies, extracted the data, and evaluated risk of bias of included studies. STATA software version 17.0 was used to conduct the network meta-analysis.

**Results:**

Our network meta-analysis included 29 studies involving 1,477 participants and 16 non-pharmacological interventions. Although most nonpharmacological interventions showed non-significant effects, the surface under the cumulative ranking curve (SUCRA) values indicated that the best non-pharmacological intervention for sleep disorders was massage therapy (97.3%), followed by music therapy (94.2%), and Treadmill training (85.7%).

**Conclusion:**

Massage therapy can be considered as an effective therapy for improving sleep quality in patients with PD. Due to limited quantity and quality of the included studies, more high quality studies are required to verify the conclusions of this network meta-analysis.

**Systematic review registration:**

identifier CRD42023429339, PROSPERO (york.ac.uk).

## 1 Introduction

Parkinson’s disease (PD), the second most common neurodegenerative disease, is a chronic senile disease. Sleep disorder is the most common non-motor symptom in PD, with an incidence of about 47.66% to 89.10% (Liu et al., 2018) and increasing year by year with the course of disease. More and more evidence shows that PD sleep disorders can lead to decreased quality of life (Zuzuárregui and During, 2020), impaired psychosocial and cognitive function (Riemann, 2019), fatigue (Cao et al., 2020), depression (Demet et al., 1999) or substance abuse (Hasler et al., 2012) and may increase the risk of cardiovascular and metabolic diseases (He et al., 2017). In addition to health risks, sleep disorders can also bring significant socio-economic burdens (Frandsen et al., 2020). Studies have shown that there are large individual differences in the manifestation of sleep disorders as assessed by questionnaires, requiring individualized treatment (Stefani and Högl, 2019). The treatment of sleep disorders includes pharmacological therapy and non-pharmacological therapy. Although many medications have been shown to have a certain therapeutic effect on sleep disorders in PD, they also have potential side effects and the overall therapeutic effect is still unsatisfactory. For example, long-term use of sedative-hypnotic drugs may lead to dependence and tolerance, and increase the risk of falls, cognitive impairment and daytime sleepiness (Zhang H. et al., 2014). Therefore, alternative non-pharmacological interventions are needed to improve sleep quality in patients with PD.

Considering the potential side effects and economic costs of pharmacological therapy, while non-pharmacological interventions has the advantages of low incidence of adverse events and strong sustainability. A wide range of non-pharmacological interventions have been used to improve sleep in patients with PD. These can be broadly categorized as follows: environmental interventions (e.g., bright light therapy), psychological interventions (e.g., cognitive behavioral therapy, mindfulness), physical activity interventions (e.g., exercise, tai chi, qigong, and yoga), physical therapy (e.g., rTMS, tDCS), and complementary and alternative therapies (e.g., music therapy, massage therapy, and acupuncture) (Paus et al., 2007; Skogar et al., 2013; Cheung et al., 2018; Wu et al., 2020; Zhu et al., 2020; Buchwitz et al., 2021; Cristini et al., 2021; Luo et al., 2021; Shan et al., 2022; Wang, 2022; Hsu et al., 2023). However, most previous studies have focused on comparing the effectiveness of single non-pharmacological intervention with usual care, sham control or waiting list in improving sleep quality in PD. There is still a lack of direct comparative studies between different non-pharmacological interventions, leading to differences in the best effectiveness of non-pharmacological interventions. For PD patients or decision-makers, they are still not known which non-pharmacological intervention is the best treatment for sleep disorders. Network meta-analysis (NMA) has been proposed to be the highest level of evidence in the treatment guideline (Salanti et al., 2014). Different from a conventional pairwise analysis, NMA analyzes simultaneously both the direct and the indirect evidence from different studies, estimation of the relative effectiveness among all interventions, and rank ordering of the interventions (Caldwell et al., 2005; Bafeta et al., 2014). The method is helpful to summarize evidence across many interventions and make optimal clinical decision (Cipriani et al., 2013). Therefore, this study used NMA to explore the effect of non-pharmacological interventions on improving sleep quality in PD, in order to provide a scientific basis for clinical medical staff to choose the optimal solution to promote sleep quality in PD patients.

## 2 Methods

### 2.1 Protocol and registration

The protocol of this NMA has been registered in PROSPERO (registration number CRD42023429339). In addition, This systematic review was performed according to the Cochrane Handbook for the Systematic Review of Interventions (Cumpston et al., 2021) and according to the Preferred Reporting Items for Systematic Reviews and Meta-Analyses (PRISMA) statement (Hutton et al., 2015). The details of the PRISMA Checklist are provided in Supplementary Table S1.

### 2.2 Search strategy

Two of the authors (RT and SG) independently searched for randomized controlled trials (RCTs) from inception to December 6, 2023 in the following databases: PubMed, Embase, the Cochrane Central Register of Controlled Trials (CENTRAL), Web of Science, China National Knowledge Infrastructure (CNKI), and Wanfang databases. A combination of Medical Subject Headings (MeSH terms or Emtree terms) and free words related to PD, non-pharmacological interventions, sleep disorders, and RCTs was used, including: (1) Parkinson disease, Parkinson’s disease, Parkinson*, paralysis agitans, PD; (2) non-pharmacolog*, intervention, treatment, training, rehabilitation, exercise, therapy, bright light therapy, BLT, repetitive transcranial magnetic stimulation, rTMS, deep brain stimulation, DBS, cognitive behavioral therapy, CBT, mindfulness meditation, Baduanjin, qigong, continuous positive airway pressure, CPAP, Tai Chi, acupuncture, massage therapy, muscle relaxation, aerobic exercise, resistance training, yoga, dance, music therapy, ultrasound therapy, low-level laser therapy, etc.; (3) dyssomnias, sleep disorders, sleep, sleepiness, sleep quality, insomnia; and (4) randomized controlled trial, randomized controlled trials as topic, controlled clinical trial, randomized, placebo. Medical Subject Headings (MeSH) and free words were linked by “OR” in each group and searched by “AND” to link each group. In addition, the reference lists of the included literature and related articles were also manually searched to identify eligible studies. The search strategies for all databases are listed in Supplementary Table S2.

### 2.3 Eligibility criteria

The PICOS (population, intervention, comparison, outcomes, study design) framework (Hutton et al., 2015) was used to operationalize the eligibility criteria of the studies to be included in the review.

A study was included if (1) population: adults (>18 years) diagnosed with PD. All participants in the intervention and control groups who were stably taking antiparkinsonian medications were also eligible, (2) intervention: participants in the experimental groups received non-pharmacological interventions with no limits in frequency, duration, style, form, or setting, (3) comparison: participants in the control groups received sham control, waiting list, or conventional treatment including usual care, supportive instruction (e.g., health education, sleep hygiene advice), and physiotherapy, or other non-pharmacological interventions that differed from the experimental group. However, original trials comparing only different approaches of the same intervention were excluded, (4) outcomes: efficacy outcomes were pre-post changes in the Pittsburgh Sleep Quality Index (PSQI), the Parkinson’s Disease Sleep Scale (PDSS), the Epworth Sleepiness Scale (ESS), the Insomnia Severity Index (ISI), the Parkinson’s disease sleep scale version 2 (PDSS-2), the Scale for Parkinson’s Disease-Sleep (SCOPA-S), and the Mini-sleep Questionnaire (MSQ). Safety outcomes were indicative of adverse events (AEs) after non-pharmacological interventions, (5) study design: only RCTs were included without any regional or publication restrictions.

Studies were excluded if they (1) cannot obtain full text or extract data; (2) were in the trial protocol registration stage and has not yet officially carried out clinical trials; (3) were conference abstracts, masteral dissertation and reviews; (4) were repeatedly published or multiple investigations were based on the same population data, the latest research or articles with comprehensive information would be included.

### 2.4 Data extraction and quality assessment

Two authors (RT and SG) independently extracted data including the first author, country, year, sample size, baseline characteristics of participants (age, gender) duration of disease, Hoehn–Yahr stage, intervention details (type, frequency, intensity and duration), comparison, and outcomes based on a predesigned form within Microsoft Excel. Two researchers (RT and SG) independently evaluated the quality according to the bias risk assessment scale of randomized controlled trials recommended by Cochrane Handbook 5.1.0 (Higgins and Green, 2011). The scale consists of seven domains: random sequence generation, allocation concealment, whether blind method was used for researchers and subjects, whether blind method was used for outcome evaluation, integrity of outcome data, selective reporting of results and other risk of bias. Each item was assessed as being of “low risk,” “high risk,” or “unclear risk” of bias. The results of the data extraction and quality assessment were cross-checked, and the divergences were resolved through discussion with a third author (CL).

### 2.5 Statistical analysis

#### 2.5.1 Traditional meta-analysis

Standard mean difference (SMD) with the corresponding 95% confidence interval (CI) was used to express the pooled estimates because all outcomes were continuous variables in this study but were measured using various tools. Due to a wide range of characteristics of the studies included, all analyses were performed using the random effects model. Statistical heterogeneity between the studies was assessed using the *I*^2^ statistic, with *I*^2^ values of 25, 50, and 75% indicating low, moderate, and high heterogeneity, respectively (Higgins et al., 2003). Moreover, subgroup analysis were used to explore the source of heterogeneity. In addition, Egger’s test were used to evaluate publication bias quantitatively (Egger et al., 1997).

#### 2.5.2 Network meta-analysis

The quality evaluation was performed using the bias risk quality evaluation tool in Review Manager 5.3. For all eligible trials, we selected the difference before and after the intervention for comparison, If the difference is not reported in the original literature, the difference is calculated according to the formula in the guide (Shi et al., 2020). Continuous variables were analyzed using SMD with 95% CI, and the significance was set at *α* = 0.05.

First, a network map of direct comparisons between different interventions was drawn by using Stata software (version 17.0). Each node in the map represents an intervention, and the size of the node indicates the sample size receiving the intervention. The presence of a line between two nodes indicates that they have a direct comparison relationship, and a thicker line indicates a higher number of comparisons. Subsequently, we examined the global consistency and used the node-split model to determine the local consistency. *p* > 0.05 indicated no significant inconsistency between direct and indirect comparisons, and in these cases, the consistency model was adopted; otherwise, the inconsistency model was used.

Additionally, the league table was used to analyze the results of the comparisons among the different interventions based on a NMA. the effects of various interventions were quantitatively analyzed by using the surface under the cumulative ranking (SUCRA) to rank the effects of different interventions. SUCRA values range from 0% to 100%, and if the SUCRA value for an intervention is closer to 100%, it indicates that the intervention is more effective. However, this conclusion should be interpreted with caution if there is not a clinically meaningful difference between the two interventions. Finally, network funnel plots were drawn and visually checked by using the symmetry criterion to determine if there was a possibility of bias leading to NMA publication.

## 3 Results

### 3.1 Study selection

**Figure 1:**
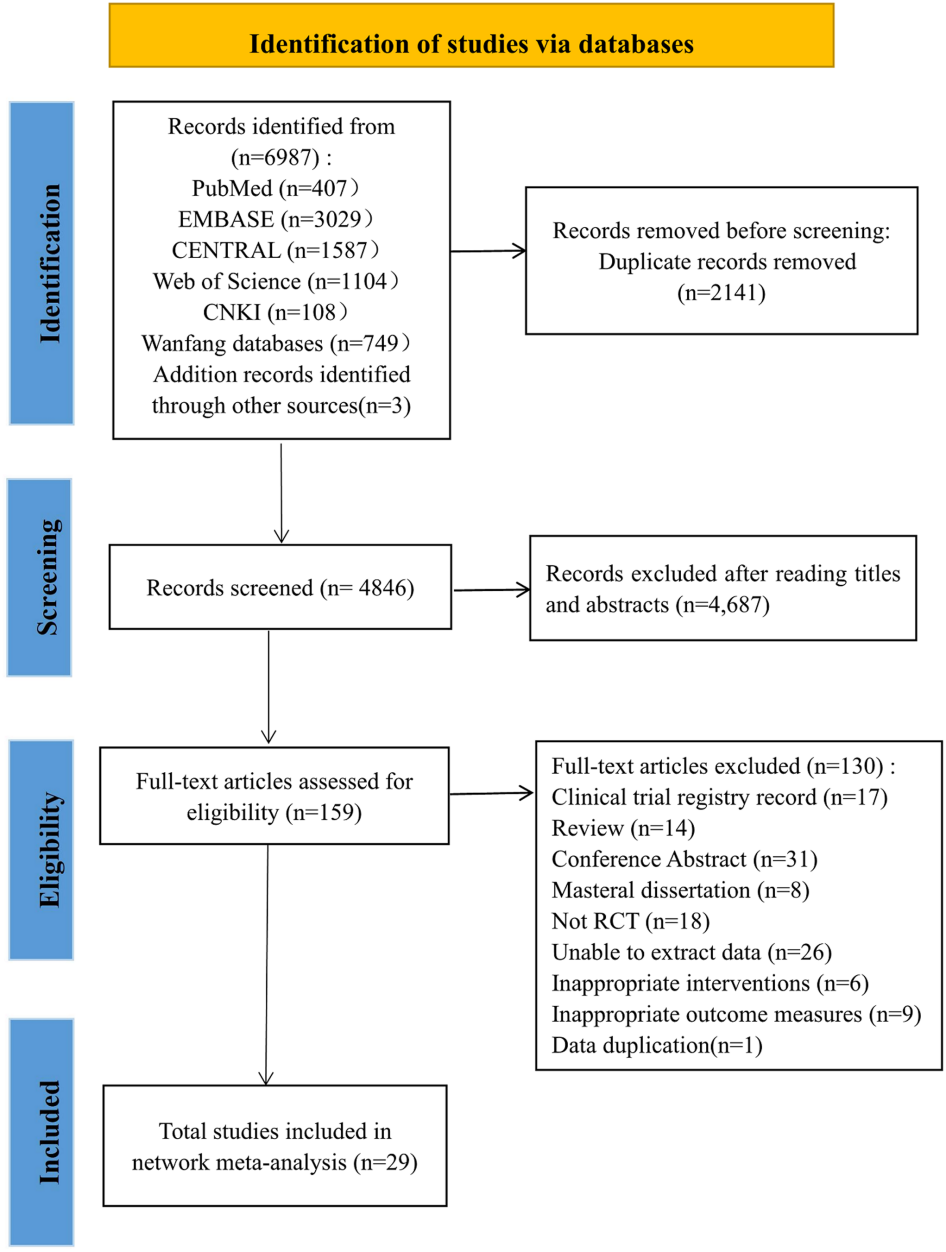
The study search, selection, eligibility and inclusion process.

Our study initially retrieved 6,987 articles, of which 2,141 were removed due to duplication, and 4,846 articles remained. Among them, 4,687 articles were excluded because their abstracts and titles did not meet the selection criteria; thus, 159 articles remained. After reading the full text, 130 articles were excluded. Finally, 29 studies were included in this study. The literature screening process was shown in Figure 1.

### 3.2 Study characteristics

Table 1 shows the characteristics of 29 eligible RCTs published from inception to 2023 and involving 1,477 participants. In this NMA, The following 16 kinds of non-pharmacological interventions were used: (1) CBT combined with BLT, (2) electroacupuncture, (3) BLT, (4) stretch-balance training, (5) aerobic exercise, (6) rTMS, (7) multimodal exercise, (8) computerized cognitive behavioral therapy, (9) tDCS, (10) baduanjin combined with qigong, (11) mindfulness, (12) qigong, (13) Tai Chi, (14) yoga, (15) music therapy, (16) massage therapy. The comparison mainly consisted of placebo, waitlist, conventional treatment. Among all eligible studies, one study was a three-arm studies (Altmann et al., 2016), and the remaining 28 studies were two-arm studies (Paus et al., 2007; Modugno et al., 2010; Nascimento et al., 2013; Rios Romenets et al., 2013; Skogar et al., 2013; Zhang W. et al., 2014; Wang et al., 2015; Xiao and Zhuang, 2015; Patel et al., 2017; Videnovic et al., 2017; Cheung et al., 2018; Rutten et al., 2019; Elyazed et al., 2020; Kraepelien et al., 2020; Moon et al., 2020; Wu et al., 2020; Xu et al., 2020; Zhu et al., 2020; Zhuang et al., 2020; Buchwitz et al., 2021; Li et al., 2021; Wu et al., 2021; Nazarova et al., 2022; Shan et al., 2022; Wang, 2022; Yu and Chen, 2022; Zhang et al., 2022; Li et al., 2023).

**Table 1:** Characteristics of included trails network meta-analysis.

References (country)	Sample (I/C)	Age (I/C)	Duration of disease, years	Hoehn–Yahr	Gender (M/F)	Intervention	Details of interventions (I)	Measured outcomes
Rios Romenets et al. (2013) (Canada)	6/6	I: 64.5 ± 16.3 C: 69.5 ± 10.5	I: 5.2 ± 1.8 C: 5.2 ± 4.4	NA	11/1	I: CBT + BLT C: sham control	CBT: 90 min each, 6 times/weeks BLT: 10,000 lux, for 30 min daily lasted 6 weeks	PSQI
Wang et al. (2015) (China)	28/20	I: 70.1 ± 6.2 C: 66.9 ± 7.9	I: 2.9 ± 2.9 C: 2.7 ± 2.3	I: 2.0 ± 0.7 C: 2.0 ± 0.8	19/26	I: electroacupuncture C: conventional treatment	30 min each, once every 3 days, lasted 8 weeks	PSQI
Videnovic et al. (2017) (America)	16/15	I: 62.31 ± 10.83 C: 64.07 ± 8.89	I: 5.94 ± 3.57 C: 8.38 ± 3.71	NA	13/18	I: BLT C: sham control	60 min each, twice a day 10,000 lux, lasted 2 weeks	PSQI
Altmann et al. (2016) (America)	11/9/10	I1:62.8 ± 8.6 I2: 63.3 ± 7.3 C: 67.8 ± 9.8	NA	NA	NA	I1: treadmill training I2: stretch-balance training C: sham control	20 min each, three times weekly, lasted 16 weeks	PSQI
Zhuang et al. (2020) (China)	19/14	I: 60.58 ± 9.21 C: 61.57 ± 13.25	I: 70.37 ± 52.26 C: 68.57 ± 45.29 (months)	I: 2 (1.5, 2.5) C: 2.25 (1.75, 3.0) Median (Q25, Q75)	18/15	I: rTMS C: sham control	1 Hz, 1,200 daily stimuli, 20 min each, intensity at 110% RMT, lasted 2 weeks	PSQI
Wu et al. (2021) (China)	49/49	I: 63.65 ± 6.02 C: 66.59 ± 8.61	I: 4.97 ± 3.91 C: 5.66 ± 3.81	NA	56/42	I: multimodal exercise C: conventional treatment	30 min each, three times weekly, lasted 8 weeks	PSQI
Li et al. (2021) (China)	50/50	I: 62.49 ± 7.53 C: 61.56 ± 7.51	I: 4.13 ± 0.58 C: 4.08 ± 0.61	NA	53/47	I: electroacupuncture C: conventional treatment	2 Hz/15 Hz, electrify: 30 min, retaining needle:30 min, 1 time/day, lasted 4 weeks	PSQI
Li et al. (2023) (China)	51/51	I: 64.17 ± 5.42 C: 64.02 ± 5.67	I: 6.17 ± 2.24 C: 6.12 ± 2.13	I: 3.24 ± 0.87 C: 3.19 ± 0.92	61/41	I: rTMS C: sham control	5 Hz, 30 min each, 1 time/day, 5 days/week, lasted 4 weeks	PSQI
Zhu et al. (2020) (China)	19/22	I: 68.53 ± 1.90 C: 67.77 ± 1.72	I: 4.68 ± 0.43 C: 4.00 ± 0.39	I: 2 ± 2.2 C: 2 ± 1.2	25/16	I: Tai Chi C: conventional treatment	30 min/time, 3 times/week, lasted 12 weeks	PDSS
Nazarova et al. (2022) (China)	19/16	I: 70.1 ± 6.2 C: 66.9 ± 7.8	NA	I: 1.6 ± 0.9 C: 2.1 ± 0.7	14/21	I: electroacupuncture C: conventional treatment	30 min each, twice weekly, lasted 8 weeks	PDSS
Xu et al. (2020) (China)	33/37	I: 61.73 ± 10.28 C: 61.95 ± 9.77	I: 3.52 ± 2.78 C: 3.26 ± 2.32	NA	36/34	I: electroacupuncture C: conventional treatment	Depth:0.8–1.5 cm, 30 min/day 4 days/week, lasted 8 weeks	PDSS
Cheung et al. (2018) (America)	10/10	I: 63.5 ± 8.5 C: 65.8 ± 6.6	4.8 ± 2.9	NA	NA	I: yoga C: waitlist	60 min each, twice weekly, lasted 12 weeks	PDSS
Skogar et al. (2013) (Sweden)	29/15	NA	NA	NA	16/28	I: massage therapy C: music therapy	60 min each, 10 times/8 weeks	PDSS
Wang (2022) (China)	38/38	I: 70.52 ± 2.32 C: 70.21 ± 1.45	NA	NA	41/35	I: music therapy C: conventional treatment	30 min each, twice a day lasted 8 weeks	PDSS
Zhang et al. (2022) (China)	15/10	I: 64.47 ± 8.44 C: 66.90 ± 10.17	NA	I: 2 (1.5, 2.5) C: 2 (2, 3) median (Q25, Q75)	15/10	I: rTMS C: sham control	60-min each, intensity of 90% of RMT, 1 Hz, 1,200 pulses each time, lasted 2 weeks	ESS
Paus et al. (2007) (Germany)	18/18	I: 63.6 ± 9.8 C: 63.4 ± 9.7	I: 7.4 ± 4.3 C: 7.9 ± 4.7	I: 2.7 ± 0.6 C: 2.5 ± 0.4	23/13	I: BLT C: sham control	7.500 lux, 30 min/every morning, 1 h/after awakening, lasted 15 days	ESS
Patel et al. (2017) (America)	14/14	I: 63.1 ± 6.8 C: 64.7 ± 9.5	NA	NA	16/12	I: CCBT C: conventional treatment	6 weeks online, interactive CBT-I	ESS
Shan et al. (2022) (China)	44/44	I: 63.48 ± 3.75 C: 61.92 ± 4.69	I: 6.12 ± 1.58 C: 5.91 ± 1.42	NA	64/24	I: rTMS C: sham control	10 Hz, intensity of 90% of RMT, 20 min/time, 5 times/week, lasted 12 weeks	ESS
Wu et al. (2020) (China)	28/26	I: 61.0 ± 11.6 C: 62.6 ± 12.2	I: 5.8 ± 2.6 C: 5.7 ± 3.5	I: 2.4 ± 0.8 C: 2.5 ± 0.6	30/24	I: tDCS C: conventional treatment	1.2 mA (stimulation intensity), 20 min/time, 5 times a week, lasted 4 weeks	ESS
Yu and Chen (2022) (China)	41/41	I: 62.62 ± 5.65 C: 62.83 ± 5.72	I: 3.08 ± 1.41 C: 3.20 ± 1.43	NA	60/22	I: rTMS C: conventional treatment	Intensity:80%–100% of RMT, 1 Hz, 20 min/time, 5 times/weeks, lasted 4 weeks	ESS
Modugno et al. (2010) (Italy)	10/10	I: 62 ± 1.58 C: 63.2 ± 1.13	I: 10 ± 1.8 C: 9.4 ± 1.1	I: 3 ± 0.22 C: 3.5 ± 0.17	10/10	I: music therapy C: conventional treatment	6 h each, 2–3 times /month lasted 3 years	ESS
Rutten et al. (2019) (Netherlands)	35/37	I: 58.9 ± 8.5 C: 65.8 ± 8.6	NA	I: 2.1 ± 0.6 C: 2.4 ± 0.7	40/32	I: BLT C: sham control	10,000 lux for 30 min; twice/day; lasted 12 weeks	SCOPA-S
Nascimento et al. (2013) (Brazil)	17/17	I: 67.8 ± 6.8 C: 66.3 ± 8.1	I: 5.1 ± 3.9 C: 4.6 ± 3.7	NA	17/17	I: multimodal exercise C: conventional treatment	60 min each, three/week, lasted 26 weeks	MSQ
Kraepelien et al. (2020) (Sweden)	38/39	I: 65.9 ± 8.5 C: 66.1 ± 9.8	I: 8.3 ± 4.4 C: 9.6 ± 5.7	NA	30/47	I: CCBT C: waitlist	One module per week (consisted of educative texts, interactive forms and a homework exercise), lasted 10 weeks	ISI
Elyazed et al. (2020) (Egypt)	15/15	I: 66.13 ± 5.66 C: 65.27 ± 4.96	NA	NA	15/15	I: treadmill training C: conventional treatment	30 min at 40%–50% of heart rate reserve (HRR); 3 times/week; lasted 12 weeks	ISI
Xiao and Zhuang (2015) (China)	48/48	I: 68.17 ± 2.27 C: 66.52 ± 2.13	I: 5.45 ± 3.61 C: 6.15 ± 2.63	I: 2.2 ± 0.21 C: 2.1 ± 0.23	67/29	I: Baduanjin + qigong C: conventional treatment	Four/week, 45 min each, lasted 26 weeks	PDSS-2
Buchwitz et al. (2021) (Germany)	14/16	I: 60.50 ± 46–79 C: 66.50 ± 54–80 [median (range)]	NA	I: 2.00 (1–3) C: 2.00 (2–3) [Median (range)]	36/35	I: mindfulness intervention C: waitlist	2 h/times, 8 times/weekly, lasted 8 weeks	PDSS-2
Moon et al. (2020) (America)	8/9	I: 66.4 ± 8.1 C: 65.9 ± 5.4	I: 4.25 ± 2.1 C: 5.33 ± 3.3	I: 2 (2-2) C: 2 (2-2) [Median (Q1–Q3)]	10/7	I: qigong C: sham control	45 min 1 h/times, 2 times/day, lasted 12 week	PDSS-2
Zhang W. et al. (2014) (China)	30/30	I: 63.73 ± 6.07 C: 65.24 ± 5.42	I: 3.93 ± 4.78 C: 4.21 ± 4.25	NA	33/27	I: rTMS C: conventional treatment	1 Hz, 30 min each, 1 time/day, 5 times/week, lasted 2 weeks	PDSS-2

I, intervention; C, control; M, male; F, female; NA, not applicable; CBT, cognitive behavioral therapy; BLT, bright light therapy; rTMS, repetitive transcranial magnetic stimulation; CCBT, computerized cognitive behavioral therapy; tDCS, transcranial direct current stimulation; PSQI, the Pittsburgh Sleep Quality Index; PDSS, the Parkinson’s Disease Sleep Scale; ESS, the Epworth Sleepiness Scale; SCOPA-S, the Scale for Parkinson’s Disease-Sleep; MSQ, the Mini-sleep Questionnaire; PDSS-2, the Parkinson’s disease sleep scale version 2; ISI, the Insomnia Severity Index.

### 3.3 Quality assessment

**Figure 2:**
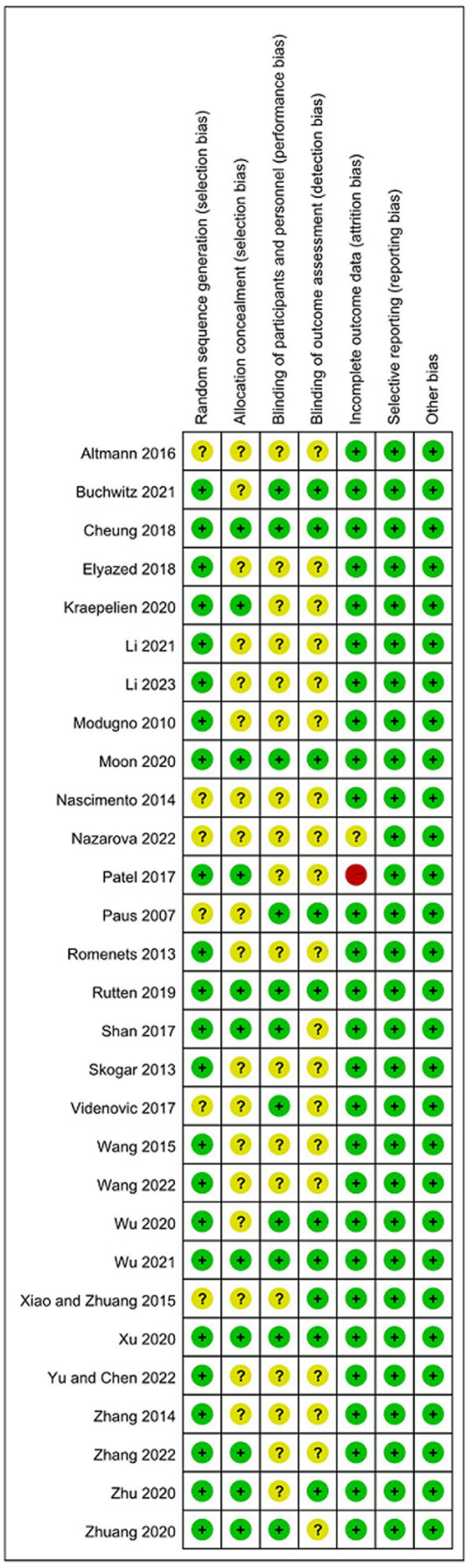
Quality assessment of the eligible studies.

Regarding the risk of bias tool, among the 29 studies included, 20 studies (Modugno et al., 2010; Rios Romenets et al., 2013; Skogar et al., 2013; Zhang W. et al., 2014; Wang et al., 2015; Cheung et al., 2018; Rutten et al., 2019; Elyazed et al., 2020; Moon et al., 2020; Wu et al., 2020; Xu et al., 2020; Zhu et al., 2020; Buchwitz et al., 2021; Li et al., 2021; Wu et al., 2021; Shan et al., 2022; Wang, 2022; Yu and Chen, 2022; Zhang et al., 2022; Li et al., 2023) mentioned the use of random number table method or software-generated random numbers for random grouping; two studies (Patel et al., 2017; Kraepelien et al., 2020) mentioned the use of envelope method for random grouping; and one study (Zhuang et al., 2020) mentioned the use of coin toss method for random grouping, all were rated as “low risk.” Six studies (Paus et al., 2007; Nascimento et al., 2013; Xiao and Zhuang, 2015; Altmann et al., 2016; Videnovic et al., 2017; Nazarova et al., 2022) only mentioned the word “random,” did not describe the randomization method, and were rated as having an “unclear risk” of bias in this field. About allocation concealment, Six studies (Patel et al., 2017; Cheung et al., 2018; Kraepelien et al., 2020; Moon et al., 2020; Xu et al., 2020; Shan et al., 2022) used sealed opaque envelopes for allocation concealment. Rutten et al. (2019) used password-protected secure drives for allocation concealment, and Wu et al. (2021) used password-protected links for allocation concealment. The remaining studies did not describe allocation concealment. Due to the characteristics of some non-drug interventions, it is difficult for researchers to implement blinding in the intervention process. Only eight studies (Videnovic et al., 2017; Cheung et al., 2018; Rutten et al., 2019; Moon et al., 2020; Wu et al., 2020; Xu et al., 2020; Buchwitz et al., 2021; Wu et al., 2021) mentioned blinding for participants and researchers, and 10 studies (Paus et al., 2007; Xiao and Zhuang, 2015; Cheung et al., 2018; Rutten et al., 2019; Moon et al., 2020; Wu et al., 2020; Xu et al., 2020; Zhu et al., 2020; Buchwitz et al., 2021; Wu et al., 2021) mentioned blinding for outcome measurers. Among the 29 studies, 20 studies had no dropout cases (Paus et al., 2007; Nascimento et al., 2013; Rios Romenets et al., 2013; Skogar et al., 2013; Zhang W. et al., 2014; Wang et al., 2015; Altmann et al., 2016; Videnovic et al., 2017; Cheung et al., 2018; Elyazed et al., 2020; Moon et al., 2020; Wu et al., 2020; Zhuang et al., 2020; Li et al., 2021; Wu et al., 2021; Shan et al., 2022; Wang, 2022; Yu and Chen, 2022; Zhang et al., 2022; Li et al., 2023), and the remaining studies had sample dropout. Among them, seven studies applied appropriate statistical analysis methods (i.e., intentional treatment analysis) (Modugno et al., 2010; Xiao and Zhuang, 2015; Rutten et al., 2019; Kraepelien et al., 2020; Xu et al., 2020; Zhu et al., 2020; Buchwitz et al., 2021), and all explained the groups from which the dropout subjects came and the specific reasons for the dropout, all were rated as “low risk.” Patel et al. (2017) described the reasons for the dropout and applied the intention-to-treat analysis, but there was a high dropout rate, so the risk of bias in this field was assessed as “unclear risk.” Nazarova et al. (2022) did not mention the causes and treatment of dropout, and was rated as having a “high risk” of bias in this field. For other biases, all studies described statistical homogeneity between groups at baseline and were rated as having a “low risk” of bias in this area. Twenty-three studies (Modugno et al., 2010; Nascimento et al., 2013; Rios Romenets et al., 2013; Wang et al., 2015; Xiao and Zhuang, 2015; Altmann et al., 2016; Patel et al., 2017; Videnovic et al., 2017; Cheung et al., 2018; Rutten et al., 2019; Kraepelien et al., 2020; Moon et al., 2020; Wu et al., 2020; Xu et al., 2020; Zhu et al., 2020; Zhuang et al., 2020; Buchwitz et al., 2021; Wu et al., 2021; Shan et al., 2022; Wang, 2022; Yu and Chen, 2022; Zhang et al., 2022; Li et al., 2023) described the approval of the Institutional Review Board (IRB). Twenty-seven studies (Modugno et al., 2010; Nascimento et al., 2013; Rios Romenets et al., 2013; Skogar et al., 2013; Zhang W. et al., 2014; Wang et al., 2015; Xiao and Zhuang, 2015; Altmann et al., 2016; Videnovic et al., 2017; Cheung et al., 2018; Rutten et al., 2019; Elyazed et al., 2020; Kraepelien et al., 2020; Moon et al., 2020; Wu et al., 2020; Xu et al., 2020; Zhu et al., 2020; Zhuang et al., 2020; Buchwitz et al., 2021; Li et al., 2021; Wu et al., 2021; Nazarova et al., 2022; Shan et al., 2022; Wang, 2022; Yu and Chen, 2022; Zhang et al., 2022; Li et al., 2023) described that they had obtained the informed consent of the participants before the experiment. The methodological quality assessments of the eligible RCTs are shown in Figure 2, ranging from low to high risk.

### 3.4 Traditional meta-analysis

#### 3.4.1 Overall effect test

The meta-analysis of the efficacy of non-pharmacological interventions to improve sleep quality in PD is shown in the forest plot in Supplementary Figure S1. The results of the combined meta-analysis showed moderate heterogeneity (*I*^2^ = 57.1%, *p* < 0.001), so the random-effects model was used to test for effect sizes. The combined effect size was (SMD: −0.47, 95% CI: −0.64, −0.30, *p* < 0.001), indicating that the combined effect size was statistically significant, i.e., non-pharmacological interventions can significantly improve sleep quality in patients with PD.

#### 3.4.2 Subgroup analysis

To further explore the sources of heterogeneity (*I*^2^ > 50%) among the studies, subgroup analysis were performed to analyze the factors causing heterogeneity. Considering the effects of different intervention durations, intervention frequencies, and intervention period on the outcomes, subgroup analyses were conducted on three factors: intervention duration, intervention frequency, and intervention period. The results of the subgroup analysis showed that among the forms of non-pharmacological interventions to improve sleep quality in patients with PD, intervention periods of ≥6 weeks and ≥5 interventions per week of <60 min are the optimal regimen to improve sleep quality in patients with PD. The results of subgroup analysis are shown in Table 2.

**Table 2:** Subgroup analysis of the effects of different covariates on sleep quality in patients with PD.

Stratified subgroups	SMD (95% CI)	*p*	*I*^2^ (%)	*p* heterogeneity
Duration of intervention				
<60 min	−0.559 (−0.775, −0.342)	<0.001	67.4	<0.001
≥60 min	−0.270 (−0.499, −0.040)	0.021	0.0	0.953
Frequency of intervention				
<5 times/weeks	−0.468 (−0.758, −0.178)	<0.001	66.1	<0.001
≥5 times/weeks	−0.507 (−0.698, −0.301)	<0.001	42.5	0.042
Intervention period				
<6 weeks	−0.416 (−0.590, −0.241)	<0.001	0.0	0.558
≥6 weeks	−0.518 (−0.758, −0.277)	<0.001	66.8	<0.001

CI, confidence interval.

### 3.5 Network meta-analysis

**Figure 3:**
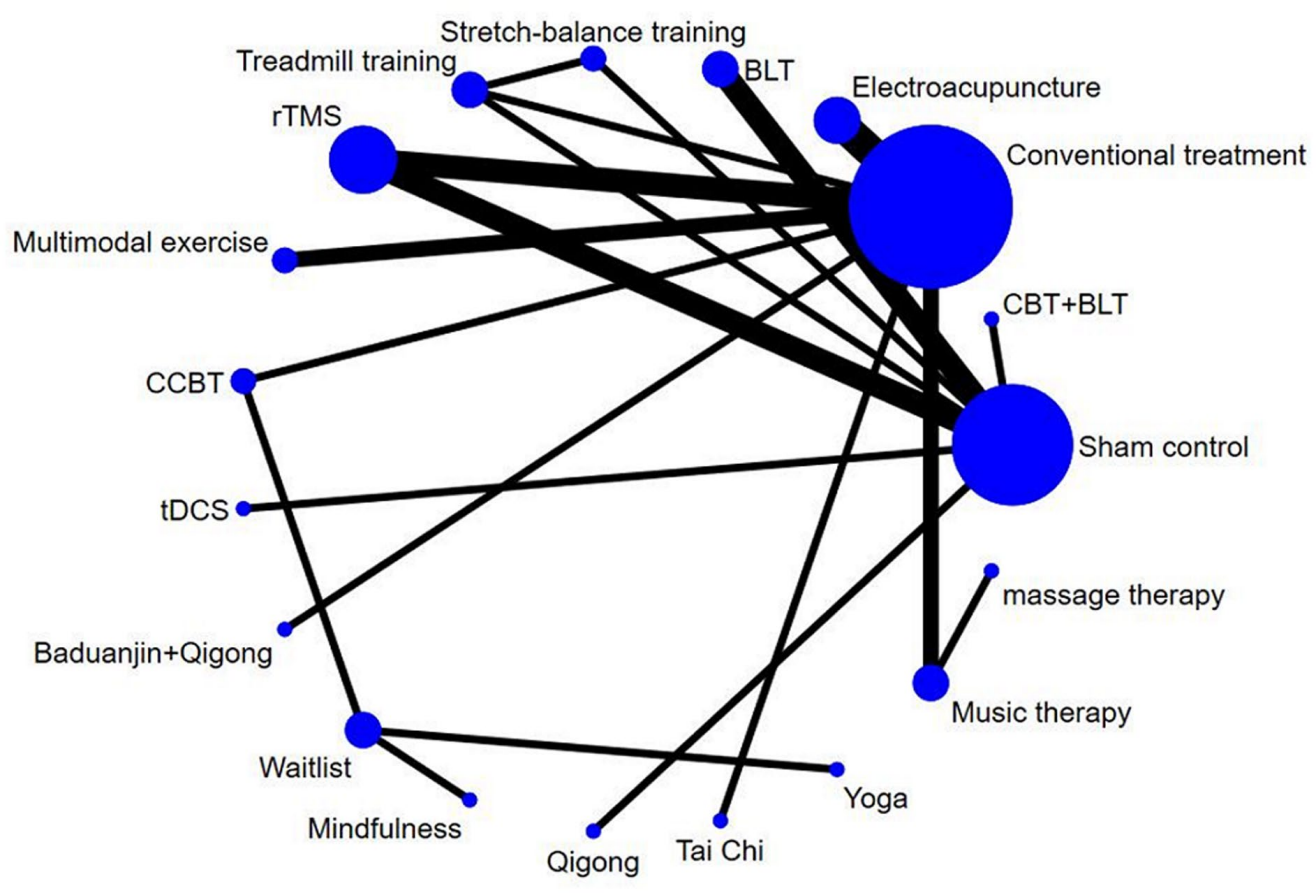
The network map of non-pharmacological interventions for sleep quality in PD. CBT, cognitive behavioral therapy; BLT, bright light therapy; rTMS, repetitive transcranial magnetic stimulation; CCBT, computerized cognitive behavioral therapy; tDCS, transcranial direct current stimulation.

The net evidence of different nonpharmacological interventions improve sleep quality in PD was shown in Figure 3. According to the network plot, rTMS, electroacupuncture, BLT, and music therapy were the more common comparisons. Sham control, treadmill training and stretch-balance training formed a closed loop, but this is a three-arm studies. Furthermore, sham control, conventional treatment, treadmill training and rTMS created a closed loop, which indicated both direct and indirect comparisons. There was no evidence of direct comparisons for the other interventions. Table 3 shows the relative effects of the different interventions on sleep quality. The league table shows the pairwise comparisons of 16 non-pharmacological interventions on sleep quality in PD.

**Table 3:** League table for non-pharmacologic interventions.

Massage therapy						
−0.23 (−0.89, 0.43)	Music therapy					
−0.68 (−1.74, 0.39)	−0.45 (−1.29, 0.40)	Treadmill training				
−1.03 (−1.94, −0.12)	−0.80 (−1.43, −0.17)	−0.35 (−1.00, 0.30)	rTMS			
−1.25 (−2.19, −0.30)	−1.02 (−1.70, −0.34)	−0.57 (−1.32, 0.18)	−0.22 (−0.69, 0.26)	Multimodal exercise		
−1.26 (−2.17, −0.34)	−1.03 (−1.66, −0.39)	−0.58 (−1.28, 0.12)	−0.23 (−0.62, 0.17)	−0.01 (−0.48, 0.46)	Electroacupuncture	
−1.28 (−2.39, −0.17)	−1.05 (−1.94, −0.15)	−0.60 (−1.48, 0.28)	−0.25 (−0.95, 0.45)	−0.03 (−0.87, 0.81)	−0.02 (−0.82, 0.78)	tDCS
−1.29 (−2.54, −0.04)	−1.06 (−2.12, −0.00)	−0.61 (−1.48, 0.25)	−0.26 (−1.17, 0.65)	−0.04 (−1.04, 0.95)	−0.03 (−1.00, 0.93)	−0.01 (−1.06, 1.03)
−1.44 (−2.81, −0.08)	−1.21 (−2.41, −0.02)	−0.77 (−1.95, 0.42)	−0.41 (−1.47, 0.64)	−0.20 (−1.35, 0.95)	−0.19 (−1.31, 0.94)	−0.17 (−1.31, 0.97)
−1.48 (−2.57, −0.39)	−1.25 (−2.12, −0.38)	−0.81 (−1.72, 0.11)	−0.45 (−1.16, 0.25)	−0.24 (−0.99, 0.52)	−0.23 (−0.93, 0.48)	−0.21 (−1.20, 0.79)
−1.55 (−3.05, −0.06)	−1.32 (−2.66, 0.02)	−0.88 (−2.21, 0.45)	−0.52 (−1.74, 0.69)	−0.31 (−1.61, 0.99)	−0.30 (−1.58, 0.98)	−0.28 (−1.57, 1.01)
−1.58 (−2.75, −0.42)	−1.35 (−2.31, −0.39)	−0.91 (−1.91, 0.10)	−0.55 (−1.37, 0.27)	−0.34 (−1.20, 0.52)	−0.33 (−1.14, 0.49)	−0.31 (−1.38, 0.77)
−1.55 (−2.54, −0.55)	−1.32 (−2.06, −0.57)	−0.87 (−1.66, −0.08)	−0.52 (−1.06, 0.03)	−0.30 (−0.90, 0.30)	−0.29 (−0.83, 0.25)	−0.27 (−1.16, 0.62)
−1.73 (−3.20, −0.25)	−1.50 (−2.81, −0.18)	−1.05 (−2.40, 0.30)	−0.70 (−1.91, 0.52)	−0.48 (−1.73, 0.77)	−0.47 (−1.69, 0.75)	−0.45 (−1.85, 0.96)
−1.66 (−2.68, −0.65)	−1.43 (−2.21, −0.66)	−0.99 (−1.74, −0.23)	−0.63 (−1.16, −0.11)	−0.42 (−1.12, 0.28)	−0.41 (−1.06, 0.25)	−0.39 (−1.07, 0.30)
−1.65 (−2.53, −0.78)	−1.42 (−2.00, −0.84)	−0.98 (−1.62, −0.33)	−0.62 (−0.90, −0.34)	−0.41 (−0.79, −0.02)	−0.40 (−0.67, −0.12)	−0.38 (−1.13, 0.38)
−1.74 (−2.69, −0.80)	−1.51 (−2.19, −0.83)	−1.07 (−1.73, −0.40)	−0.71 (−1.10, −0.32)	−0.50 (−1.10, 0.11)	−0.49 (−1.04, 0.06)	−0.47 (−1.05, 0.11)
−2.00 (−3.56, −0.45)	−1.77 (−3.18, −0.36)	−1.33 (−2.76, 0.11)	−0.97 (−2.29, 0.34)	−0.76 (−2.10, 0.59)	−0.74 (−2.06, 0.57)	−0.73 (−2.22, 0.77)
−2.04 (−3.31, −0.77)	−1.81 (−2.89, −0.73)	−1.36 (−2.48, −0.24)	−1.01 (−1.97, −0.05)	−0.79 (−1.79, 0.20)	−0.78 (−1.74, 0.17)	−0.76 (−1.95, 0.42)

Stretch-balance training						
−0.15 (−1.47, 1.16)	Qigong					
−0.19 (−1.32, 0.94)	−0.04 (−1.31, 1.23)	Tai Chi				
−0.26 (−1.71, 1.18)	−0.11 (−1.63, 1.40)	−0.07 (−1.48, 1.34)	CBT + BLT			
−0.29 (−1.50, 0.91)	−0.14 (−1.48, 1.20)	−0.10 (−1.11, 0.91)	−0.03 (−1.50, 1.44)	CCBT		
−0.26 (−1.29, 0.78)	−0.10 (−1.29, 1.09)	−0.06 (−0.86, 0.74)	0.01 (−1.33, 1.34)	0.04 (−0.86, 0.93)	Baduanjin + Qigong	
−0.43 (−1.94, 1.07)	−0.28 (−1.89, 1.33)	−0.24 (−1.59, 1.11)	−0.17 (−1.89, 1.55)	−0.14 (−1.05, 0.76)	−0.18 (−1.45, 1.09)	Mindfulness
−0.37 (−1.32, 0.57)	−0.22 (−1.27, 0.83)	−0.18 (−1.06, 0.70)	−0.11 (−1.32, 1.10)	−0.08 (−1.06, 0.89)	−0.12 (−0.88, 0.64)	0.06 (−1.27, 1.39)
−0.36 (−1.29, 0.57)	−0.21 (−1.30, 0.89)	−0.17 (−0.82, 0.48)	−0.10 (−1.35, 1.15)	−0.07 (−0.84, 0.70)	−0.11 (−0.57, 0.36)	0.07 (−1.11, 1.26)
−0.45 (−1.32, 0.42)	−0.30 (−1.28, 0.68)	−0.26 (−1.07, 0.55)	−0.19 (−1.34, 0.97)	−0.16 (−1.07, 0.75)	−0.20 (−0.87, 0.47)	−0.02 (−1.30, 1.26)
−0.71 (−2.29, 0.87)	−0.56 (−2.24, 1.13)	−0.52 (−1.96, 0.92)	−0.45 (−2.24, 1.35)	−0.42 (−1.45, 0.61)	−0.45 (−1.82, 0.91)	−0.28 (−1.45, 0.90)
−0.75 (−2.05, 0.55)	−0.60 (−2.02, 0.83)	−0.56 (−1.68, 0.57)	−0.49 (−2.04, 1.07)	−0.46 (−0.96, 0.04)	−0.49 (−1.52, 0.53)	−0.31 (−1.07, 0.44)

BLT				
0.01 (−0.59, 0.61)	Conventional treatment			
−0.08 (−0.44, 0.28)	−0.09 (−0.57, 0.39)	Sham control		
−0.34 (−1.75, 1.08)	−0.35 (−1.63, 0.94)	−0.26 (−1.63, 1.11)	Yoga	
−0.38 (−1.47, 0.72)	−0.39 (−1.30, 0.53)	−0.30 (−1.33, 0.74)	−0.04 (−0.94, 0.86)	Waitlist

rTMS, repetitive transcranial magnetic stimulation; tDCS, transcranial direct current stimulation; CBT, cognitive behavioral therapy; BLT, bright light therapy; CCBT, computerized cognitive behavioral therapy.

### 3.6 Rank probability

**Figure 4:**
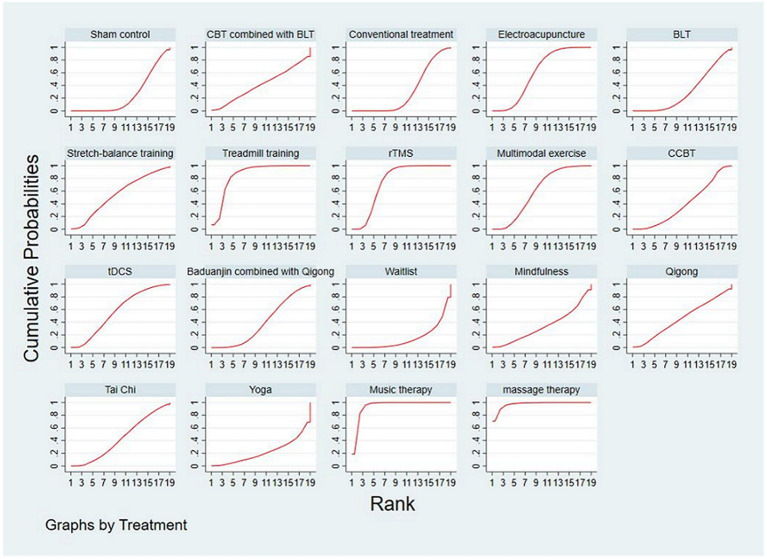
The SUCRA plot based on cumulative probabilities of interventions. CBT, cognitive behavioral therapy; BLT, bright light therapy; rTMS, repetitive transcranial magnetic stimulation; CCBT, computerized cognitive behavioral therapy; tDCS, transcranial direct current stimulation.

The SUCRA plot and values are shown in Figure 4 and Table 4, respectively. The SUCRA values and the plot revealed that the treatments’ comparative efficacy in improving sleep quality was, in order: massage therapy > music therapy > treadmill training > rTMS > multimodal exercise > electroacupuncture > tDCS > stretch-balance training > qigong > Tai Chi > CBT combined with BLT > CCBT > baduanjin combined with qigong > mindfulness > BLT > conventional treatment > sham control > yoga > waitlist.

**Table 4:** SUCRA values for non-pharmacologic interventions.

Treatments	SUCRA
Sham control	24.4
CBT combined with BLT	41.0
Conventional treatment	30.3
Electroacupuncture	60.2
BLT	31.1
Stretch-balance training	54.9
Treadmill training	85.7
rTMS	74.6
Multimodal exercise	60.2
CCBT	39.8
tDCS	57.1
Baduanjin combined with Qigong	39.2
Waitlist	14.8
Mindfulness	34.0
Qigong	45.8
Tai Chi	43.5
Yoga	21.9
Music therapy	94.2
Massage therapy	97.3

CBT, cognitive behavioral therapy; BLT, bright light therapy; rTMS, repetitive transcranial magnetic stimulation; CCBT, computerized cognitive behavioral therapy; tDCS, transcranial direct current stimulation.

### 3.7 Consistency analysis

The global inconsistency analysis of this NMA showed a p-value of 0.267, indicating no significant inconsistency. The results are summarized in Supplementary Figure S2. Moreover, the results of the node-splitting analysis showed no inconsistency between direct and indirect comparisons (*p* = 0.267 to 0.996). The results are summarized in Supplementary Table 3A. Therefore, we used the consistency model to perform the NMA. Sham control-conventional treatment-aerobic exercise-rTMS formed a quadratic loops without significant discordance (IF = 0.674, 95% CI = 0.00–2.13, tau^2^ = 0.034). The multi-arm trials formed a triangular loop: Sham control-aerobic exercise-stretch-balance training, which was defined as no inconsistency.

### 3.8 Publication analysis

**Figure 5:**
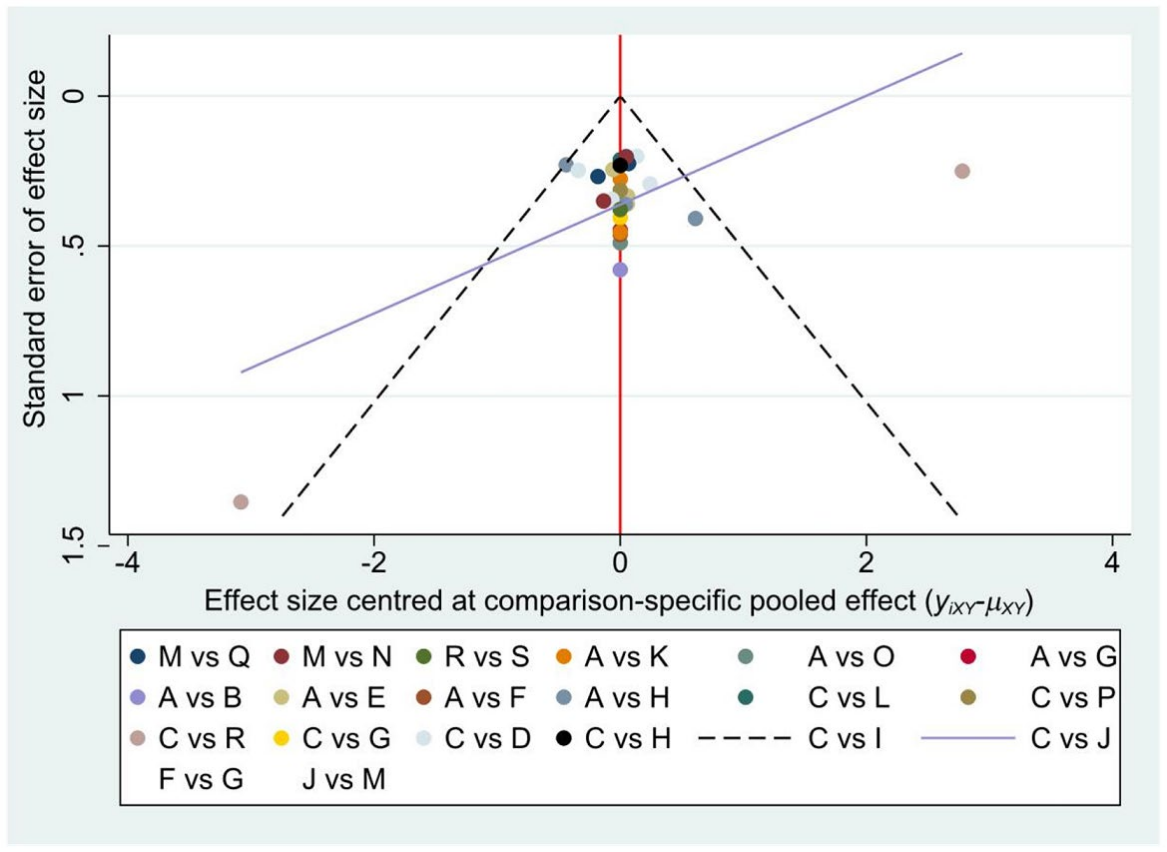
The funnel plot of non-pharmacological interventions for sleep quality in PD.

To assess publication bias, a comparison-adjusted network funnel plot with a random model was constructed for the outcome (Figure 5). The results were roughly distributed around the overall effect, arranged symmetrically around the center line. The included literature was well distributed, the data analysis results were less affected by the publication bias. Egger’s test also showed no publication bias (*p* = 0.269) (Supplementary Table 3B).

### 3.9 Incidence of adverse events

For the incidence of AEs, quantitative synthesis was judged to be inadequate because the number of AEs and the number of patients experiencing AEs were mixed, so qualitative analysis is adopted. Thirteen studies (Paus et al., 2007; Rios Romenets et al., 2013; Videnovic et al., 2017; Rutten et al., 2019; Kraepelien et al., 2020; Zhu et al., 2020; Nazarova et al., 2022; Yu and Chen, 2022) reported the incidences of AEs, with five studies (Cheung et al., 2018; Xu et al., 2020; Zhuang et al., 2020; Buchwitz et al., 2021; Zhang et al., 2022) reporting no AEs. In Yu and Chen (2022), there was one case of headache, one case of drowsiness, two cases of memory loss in the ATU group, while there were two cases of headache in the rTMS group, but their symptoms were relieved quickly. Rutten et al. reported mild ocular symptoms, headache, and gastrointestinal distress (Rutten et al., 2019). Rios Romenets et al. (2013) reported that one patient in the intervention group fell asleep while using BLT, resulting in mild facial burns with no serious consequences. In Videnovic et al. (2017), there were two cases of headache and sleepiness occurred in the BLT group, while there was one case of itchy eyes occurred in the placebo group, and these adverse effects subsided spontaneously. In Paus et al. (2007), four patients in the intervention group reported minor and transitory side effects, being eyestrain, and feeling of general malaise (two patients each, respectively). In Kraepelien et al. (2020), there were one patient experienced temporary stomach discomfort during meditation practice and felt depressed. In Zhu et al. (2020), two participants in the Tai Chi group and one participant in the conventional treatment group reported fatigue and dizziness, and there was one case of muscle spasm in each group. In Nazarova et al. (2022), there was one case of fall in the acupuncture group.

## 4 Discussion

A total of 29 RCT studies of non-pharmacological interventions to improve sleep quality in PD were included in this study, and the included studies were subjected to rigorous quality evaluation and risk assessment. To the best of our knowledge, this is the first NMA comparing the efficacy of different non-pharmacologic interventions for improving sleep quality in PD. The SUCRA values revealed that the best non-pharmacological intervention was massage therapy, followed by music therapy and treadmill training. Subgroup analyses further indicated that non-pharmacological interventions of ≥6 weeks duration, ≥5 interventions per week, and less than 60 min per intervention were the best regimen for improving sleep quality in patients with PD. Furthermore, In terms of the incidence of AEs, all non-pharmacologic interventions resulted in few and mild AEs, improving the clinical safety of the treatment and increasing the compliance of patients.

Sleep disorders in PD are caused by a variety of factors, including the degeneration of central sleep regulation, disease progression, medications, comorbidities, and mood disorders (Gunn et al., 2010; Rothman and Mattson, 2012; Falup-Pecurariu and Diaconu, 2017) particularly, motor symptoms of PD, such as nocturnal hypokinesia, rigidity, tremor exacerbated by overnight wearing-off and painful dystonia, may contribute to sleep onset and/or sleep maintenance difficulties (Stack and Ashburn, 2006; Louter et al., 2013). In addition, nonmotor symptoms such as nocturia, pain, depression, anxiety and hallucinations, often lead to sleep fragmentation (Gómez-Esteban et al., 2006; Wailke et al., 2010; Yong et al., 2011). Various non-pharmacological interventions, as alternative or complementary therapies, can consistently and effectively improve motor and nonmotor symptoms in PD, leading to improved sleep quality.

Existing evidence suggests that massage therapy improves sleep quality in different populations (Pruthi et al., 2009; Nerbass et al., 2010). In addition, our findings suggest that massage therapy is the best non-pharmacologic intervention for improving sleep quality in PD with a SUCRA value of 97.3%. Massage therapy refers to a technical operation by pressing, moistening, pushing, holding, kneading and other techniques on specific parts of the human body surface, easy to operate, safe and non-invasive, high acceptance of patients. Moderate pressure applied during massage may enhance vagal activity and inhibit hypothalamic-pituitary-adrenal function by stimulating pressure receptors that eventually signal the limbic system, which in turn leads to lower norepinephrine and cortisol levels and higher serotonin levels (Field, 2016). Decreased norepinephrine levels slow heart rate, lower blood pressure, and improve sleep (Blagrove et al., 2012). Decreased cortisol levels are thought to be associated with improved sleep disorders such as obstructive sleep apnea and insomnia (Blagrove et al., 2012). Increased serotonin levels may improve depressive symptoms, relieve pain (Field, 2016), and thus improve overall sleep quality. In addition, massage therapy transmits stimulation to the autonomic nervous system, activates blood circulation, relieves muscle tension, releases stress, relaxes the body and promotes sleep. However, the number of studies on the effects of massage therapy on sleep quality in PD is limited, and the results of the present study were derived from only one RCT study (Skogar et al., 2013), which had a small sample size and poor methodological quality, limiting the accuracy and generalizability of the conclusions, and the results should be treated with caution. Therefore, more high-quality, large-sample size RCTs are needed in the future to validate the long-term effects of massage therapy on sleep quality in PD.

Music is one of the most-used self-help strategies to promote sleep. A Cochrane review (Jespersen et al., 2015) showed that music therapy was effective in improving subjective sleep quality in adult insomnia patients. Although the causes and mechanisms are unknown, the effects of music therapy on sleep quality can be explained by the following reasons. Firstly, music’s ability to regulate emotional states may help improve patients’ sleep quality. The study showed that listening to music that participants experience as pleasurable elicit increased activity in the mesolimbic reward network of the brain including the dorsal and ventral striatum (Jespersen et al., 2023). Secondly, music is closely related to physiological responses, sedative music induces a relaxation and distraction response, which reduces activity in the neuroendocrine and sympathetic nervous systems, resulting in decreased pain, stress, anxiety, and sleep (Jespersen et al., 2015). In addition, other mechanisms may come into play such as the masking effect of music whereby music facilitates sleep by covering noise from the hospital environment (Jespersen et al., 2023). Our study showed that music therapy ranked second among nonpharmacological interventions with a SUCRA value of 94.2% and was effective in improving sleep quality in patients with PD. However, the relationship between the objective characteristics of music and the subjective preferences of individuals remains unclear, and more RCTs are needed in the future to investigate potential differences in the effects of researcher-selected music and participant-selected music.

Previous systematic reviews have only demonstrated the efficacy of exercise on sleep quality in PD (Cristini et al., 2021), there have been no cross-sectional comparisons for different exercise types, so it would become meaningful to explore comparisons of efficacy between them. Our study showed that treadmill training was the third ranked non-pharmacological intervention with a SUCRA value of 85.7%. Treadmill training, one of the most common forms of aerobic exercise, promotes increased levels of brain-derived neurotrophic factor (BDNF) and induces sleep by creating a state of energy expenditure and increasing basal metabolic rate (Doris et al., 2018). Moderate-intensity treadmill training also reduces resting plasma concentrations of pro-inflammatory cytokines and increases plasma concentrations of anti-inflammatory cytokines, thereby improving sleep quality (Kapsimalis et al., 2008). Sacheli et al. has been confirmed that three-month treadmill training can increase cortico-striatal neuroplasticity and dopamine release to improve sleep quality (Sacheli et al., 2019). In addition, treadmill training is not limited by road conditions, weather, and time, and is suitable for any age and any place, which can stimulate the potential of patients and achieve lasting and stable therapeutic effects (Schootemeijer et al., 2020). In short, treadmill training can be considered an important method to assist in the treatment of sleep disorders in PD.

Our study showed that rTMS can improve sleep quality in PD, which is consistent with the results of previous studies. rTMS is a noninvasive neurostimulation technique with the advantages of noninvasiveness, painlessness, high therapeutic safety, precise clinical efficacy, and few adverse reactions (Yang et al., 2018). rTMS can induce hyperpolarization of cortical neurons, inhibit the excitability and metabolic level of cerebral cortex, and improve sleep state (Hsu et al., 2007). Song et al. (2019) concluded that rTMS can promote the synthesis of pineal gland and secreting melatonin in PD patients, thus effectively regulating the sleep cycle (Gorfine and Zisapel, 2007). And rTMS can regulate the levels of various neurotransmitters (5-HT, NE, DA) and maintain the balance of neurotransmitters in the body, thus improving sleep quality (Kiebs et al., 2019). Due to the small number of included articles, our study did not perform a subgroup analysis of the stimulation frequency of rTMS (e.g., 1 Hz/5 HZ) to further explore the efficacy of different stimulation frequencies on sleep quality in PD. Therefore, more large sample, high quality RCTs are needed in the future to explore the optimal stimulation parameters and stimulation modes for rTMS to improve sleep quality in PD.

Multimodal exercise refers to the intervention of two or more types of exercise (Suzuki et al., 2012), such as aerobic exercise, strength/resistance training, balance/coordination training, flexibility training and other combinations of exercise methods. Its rich environment and exercise types can induce more new neurons and delay disease progression (Hirsch and Farley, 2009; Chirles et al., 2017). Several studies have shown that multimodal exercise can improve muscle strength, flexibility and balance, effectively improve motor disorders and ADL in PD patients, reduce depression symptoms, and improve the overall sleep quality of patients with PD (Suzuki et al., 2012; Vogel et al., 2021). It is worth noting that multimodal exercise as a combination therapy should be more effective than treadmill training, but this was not the case in our results. The reason for this effect may be that PD patients are mostly elderly patients with severe functional impairment, and even most of them have cognitive dysfunction, which makes it difficult for them to understand and perform complex, high-intensity multimodal exercise, and thus patients’ motivation and exercise compliance are poor, resulting in poor quality of exercise and difficulty in achieving the desired rehabilitation effect (Schootemeijer et al., 2020).

Traditional meta-analyses have shown that electroacupuncture significantly improves sleep quality (Hsu et al., 2023), which is consistent with the results of this study. Electroacupuncture has a weaker effect on insomnia itself, but it shows excellent results in improving accompanying symptoms, especially pain and pain-related insomnia. However, standardized acupoint selection protocols are a major barrier to the spread of acupuncture therapy (Hsu et al., 2023). In addition, our findings showed that the remaining non-pharmacological interventions had no significant effect on sleep disorders in PD. This may be due to the insufficient number of RCTs included in these interventions, small sample size and/or disease progression (Rothman and Mattson, 2012; Louter et al., 2013; Cheung et al., 2018; Liu et al., 2018; Moon et al., 2020; Zhu et al., 2020; Buchwitz et al., 2021). In particular, the relevant guidelines recommend CBT as a first-level recommended treatment for insomnia in the elderly (Koychev and Okai, 2017), but there are few RCTs on the application of CBT to PD sleep disorders, and the evidence is relatively weak. Therefore, readers should be cautious about these results. In the future, more RCTs are needed to verify the efficacy of these non-pharmacological intervention strategies for sleep disorders in PD.

Our study also had some limitations. Firstly, there was heterogeneity in the included studies, such as the duration, frequency, and period of non-pharmacologic interventions. Secondly, we comprehensively searched for non-pharmacological interventions for sleep disorders in PD, but the language was limited to Chinese and English, which may have contributed to selection bias. Thirdly, many comparisons between non-pharmacological interventions include only a small number of RCTs, which may have affected the accuracy of the conclusions. Fourthly, most of the studies included in this network meta-analysis compared nonpharmacological therapies with conventional treatment, placebo or wait-list, while the actual number of head-to-head trials was relatively small, so efficacy comparisons between interventions are often based on indirect comparisons. Finally, the overall quality of most RCTs included in this study was moderate, especially due to the lack of blinding procedures, there was some risk of overestimation. Due to the nature of non-pharmacological interventions, blinding of participants and researchers seems inevitable. In the future, higher quality research is needed to avoid or reduce the risk of overestimation.

## 5 Conclusion

Our study compared the efficacy of different non-pharmacological interventions for sleep disorders in patients with PD, and our results suggest that massage therapy may be the preferred non-pharmacological intervention for improving sleep disorders in PD, followed by music therapy and treadmill training. However, the results should be interpreted with caution, considering the limitations of our above meta-analysis and the limited number of existing RCT and the small sample size, other potential risks of bias (e.g. lack of blinded procedures), and some differences in study design.

Therefore, further validation with more large sample, high-quality RCTs are needed to ensure the scientificity of the findings. Finally, the results of this study could provide evidence and a reference to healthcare providers and clinicians when choosing effective interventions to improve the quality of life and health status of patients with PD.

## Data availability statement

The original contributions presented in the study are included in the article/Supplementary material, further inquiries can be directed to the corresponding authors.

## Author contributions

RT: Data curation, Methodology, Software, Writing – original draft, Writing – review & editing. SG: Data curation, Software, Writing – review & editing. JLi: Data curation, Writing – review & editing. WH: Data curation, Writing – review & editing. JLiu: Conceptualization, Supervision, Writing – review & editing. CL: Conceptualization, Supervision, Writing – review & editing.

## References

[ref1] Altmann L. J. P. Stegemöller E. Hazamy A. A. Wilson J. P. Bowers D. Okun M. S. . (2016). Aerobic exercise improves mood, cognition, and language function in Parkinson’s disease: results of a controlled study. J. Int. Neuropsychol. Soc. 22, 878–889. doi: 10.1017/s135561771600076x, PMID: 27655232

[ref2] Bafeta A. Trinquart L. Seror R. Ravaud P. (2014). Reporting of results from network meta-analyses: methodological systematic review. BMJ 348:1741. doi: 10.1136/bmj.g1741, PMID: 24618053 PMC3949412

[ref3] Blagrove M. Fouquet N. C. Baird A. L. Pace-Schott E. F. Davies A. C. Neuschaffer J. L. . (2012). Association of salivary-assessed oxytocin and cortisol levels with time of night and sleep stage. J. Neural Transm. 119, 1223–1232. doi: 10.1007/s00702-012-0880-1, PMID: 22911329

[ref4] Buchwitz T. M. Maier F. Greuel A. Thieken F. Steidel K. Jakobs V. . (2021). Pilot study of mindfulness training on the self-awareness of motor symptoms in Parkinson’s disease-a randomized controlled trial. Front. Psychol. 12:763350. doi: 10.3389/fpsyg.2021.763350, PMID: 34916997 PMC8670006

[ref5] Caldwell D. M. Ades A. E. Higgins J. P. T. (2005). Simultaneous comparison of multiple treatments: combining direct and indirect evidence. BMJ 331, 897–900. doi: 10.1136/bmj.331.7521.897, PMID: 16223826 PMC1255806

[ref6] Cao X.-Y. Zhang J.-R. Shen Y. Mao C. J. Shen Y. B. Cao Y. L. . (2020). Fatigue correlates with sleep disturbances in Parkinson disease. Chin. Med. J. 134, 668–674. doi: 10.1097/cm9.0000000000001303, PMID: 33725706 PMC7990014

[ref7] Cheung C. Bhimani R. Wyman J. F. Konczak J. Zhang L. Mishra U. . (2018). Effects of yoga on oxidative stress, motor function, and non-motor symptoms in Parkinson’s disease: a pilot randomized controlled trial. Pilot Feasibility Stud. 4:162. doi: 10.1186/s40814-018-0355-8, PMID: 30377537 PMC6198358

[ref8] Chirles T. J. Reiter K. Weiss L. R. Alfini A. J. Nielson K. A. Smith J. C. (2017). Exercise training and functional connectivity changes in mild cognitive impairment and healthy elders. J. Alzheimers Dis. 57, 845–856. doi: 10.3233/JAD-161151, PMID: 28304298 PMC6472271

[ref9] Cipriani A. Higgins J. P. T. Geddes J. R. Salanti G. (2013). Conceptual and technical challenges in network meta-analysis. Ann. Intern. Med. 159, 130–137. doi: 10.7326/0003-4819-159-2-201307160-00008, PMID: 23856683

[ref10] Cristini J. Weiss M. De Las Heras B. Medina-Rincón A. Dagher A. Postuma R. B. . (2021). The effects of exercise on sleep quality in persons with Parkinson’s disease: a systematic review with meta-analysis. Sleep Med. Rev. 55, 101–384. doi: 10.1016/j.smrv.2020.101384, PMID: 32987321

[ref11] Cumpston M. Li T. Page M. J. Chandler J. Welch V. A. Higgins J. P. . (2021). Updated guidance for trusted systematic reviews: a new edition of the Cochrane handbook for systematic reviews of interventions. Cochrane Database Syst. Rev. 10:ED000142. doi: 10.1002/14651858.ed000142, PMID: 31643080 PMC10284251

[ref12] Demet M. E. Chicz-Demet A. Fallon J. H. Sokolski K. N. (1999). Sleep deprivation therapy in depressive illness and Parkinson’s disease. Prog. Neuropsychopharmacol. Biol. Psychiatry 23, 753–784. doi: 10.1016/s0278-5846(99)00039-1, PMID: 10509373

[ref13] Doris Yu S. F. Ng S. S. M. Lee D. T. F. Choi K. C. Siu P. M. F. . (2018). The effects of an activity-based lifestyle intervention on moderate sleep complaints among older adults: study protocol for a randomized controlled trial. Trials 19:69. doi: 10.1186/s13063-018-2465-2, PMID: 29370818 PMC5785807

[ref14] Egger M. Smith G. D. Schneider M. Minder C. (1997). Bias in meta-analysis detected by a simple, graphical test. BMJ 315, 629–634. doi: 10.1136/bmj.315.7109.629, PMID: 9310563 PMC2127453

[ref15] Elyazed T. I. A. Al-Azab I. M. A. Semary M. M. E. (2020). Effect of aerobic exercise on depression and insomnia in Egyptian geriatrics Parkinson’s population. Biosci. Res. 15, 1601–1609.

[ref16] Falup-Pecurariu C. Diaconu S. (2017). Sleep dysfunction in Parkinson’s disease. Int. Rev. Neurobiol. 133, 719–742. doi: 10.1016/bs.irn.2017.05.033 28802939

[ref17] Field T. (2016). Massage therapy research review. Complement. Ther. Clin. Pract. 24, 19–31. doi: 10.1016/j.ctcp.2016.04.005, PMID: 27502797 PMC5564319

[ref18] Frandsen R. Asah C. Ibsen R. Kjellberg J. Jennum P. J. (2020). Health, social, and economic consequences of rapid eye movement sleep behavior disorder: a controlled national study evaluating societal effects. Sleep 44:zsaa162. doi: 10.1093/sleep/zsaa162, PMID: 32844211

[ref19] Gómez-Esteban J. C. Zarranz J. J. Lezcano E. Velasco F. Ciordia R. Rouco I. . (2006). Sleep complaints and their relation with drug treatment in patients suffering from Parkinson’s disease. Mov. Disord. 21, 983–988. doi: 10.1002/mds.20874, PMID: 16602112

[ref20] Gorfine T. Zisapel N. (2007). Late evening brain activation patterns and their relation to the internal biological time, melatonin, and homeostatic sleep debt. Hum. Brain Mapp. 30, 541–552. doi: 10.1002/hbm.20525, PMID: 18095278 PMC6871121

[ref21] Gunn D. G. Naismith S. L. Lewis S. J. G. (2010). Sleep disturbances in Parkinson disease and their potential role in heterogeneity. J. Geriatr. Psychiatry Neurol. 23, 131–137. doi: 10.1177/0891988709358591, PMID: 20101072

[ref22] Hasler B. P. Smith L. J. Cousins J. C. Bootzin R. R. (2012). Circadian rhythms, sleep, and substance abuse. Sleep Med. Rev. 16, 67–81. doi: 10.1016/j.smrv.2011.03.004, PMID: 21620743 PMC3177010

[ref23] He Q. Zhang P. Li G. Dai H. Shi J. (2017). The association between insomnia symptoms and risk of cardio-cerebral vascular events: a meta-analysis of prospective cohort studies. Eur. J. Prev. Cardiol. 24, 1071–1082. doi: 10.1177/2047487317702043, PMID: 28359160

[ref24] Higgins J. P. T. Green S. (2011). Cochrane handbook for systematic reviews of interventions. 2nd Edn. Chichester, United Kingdom: John Wiley & Sons.

[ref25] Higgins J. P. T. Thompson S. G. Deeks J. J. Altman D. G. (2003). Measuring inconsistency in meta-analyses. BMJ 327, 557–560. doi: 10.1136/bmj.327.7414.557, PMID: 12958120 PMC192859

[ref26] Hirsch M. A. Farley B. G. (2009). Exercise and neuroplasticity in persons living with Parkinson’s disease. Eur. J. Phys. Rehabil. Med. 45, 215–229 19532109

[ref27] Hsu W. Y. Hsu C.-M. Hung S.-C. Hung S.-Y. (2023). Acupuncture improves sleep disorders and depression among patients with Parkinson’s disease: a meta-analysis. Healthcare 11:2042. doi: 10.3390/healthcare11142042, PMID: 37510483 PMC10379076

[ref28] Hsu J.-L. Jung T.-P. Hsu C.-Y. Hsu C. Y. Hsu W. C. Chen Y. K. . (2007). Regional CBF changes in Parkinson’s disease: a correlation with motor dysfunction. Eur. J. Nucl. Med. Mol. Imaging 34, 1458–1466. doi: 10.1007/s00259-006-0360-7, PMID: 17437108

[ref29] Hutton B. Salanti G. Caldwell D. M. Chaimani A. Schmid C. H. Cameron C. . (2015). The PRISMA extension statement for reporting of systematic reviews incorporating network meta-analyses of health care interventions: checklist and explanations. Ann. Intern. Med. 162, 777–784. doi: 10.7326/m14-2385, PMID: 26030634

[ref30] Jespersen K. V. Hansen M. H. Vuust P. (2023). The effect of music on sleep in hospitalized patients: a systematic review and meta-analysis. Sleep Health 9, 441–448. doi: 10.1016/j.sleh.2023.03.004, PMID: 37380591

[ref31] Jespersen K. V. Koenig J. Jennum P. Vuust P. Cochrane Developmental, Psychosocial and Learning Problems Group (2015). Music for insomnia in adults. Cochrane Database Syst. Rev. 2015:CD010459. doi: 10.1002/14651858.cd010459.pub2, PMID: 26270746 PMC9241357

[ref32] Jun Y. Yongzheng W. Caiqiong L. (2022). Clinical efficacy of high frequency rTMS combined with selegilan hydrochloride in the treatment of PD with excessive daytime sleepiness sleep disorder and its correlation with DTI. Chin. J. Rehabil. 37, 592–597. doi: 10.3870/zgkf.2022.10.004

[ref33] Kapsimalis F. Basta M. Varouchakis G. Gourgoulianis K. Vgontzas A. Kryger M. (2008). Cytokines and pathological sleep. Sleep Med. 9, 603–614. doi: 10.1016/j.sleep.2007.08.019 18024171

[ref34] Kelmanson I. A. Adulas E. I. (2006). Massage therapy and sleep behavior in infants born with low birth weight. Complement. Ther. Clin. Pract. 12, 200–205. doi: 10.1016/j.ctcp.2005.11.007, PMID: 16835031

[ref35] Kiebs M. Hurlemann R. Mutz J. (2019). Repetitive transcranial magnetic stimulation in non-treatment-resistant depression. Br. J. Psychiatry 215, 445–446. doi: 10.1192/bjp.2019.75, PMID: 31014413

[ref36] Koychev I. Okai D. (2017). Cognitive-behavioral therapy for non-motor symptoms of Parkinson’s disease: a clinical review. Evid. Based Ment. Health 20, 15–20. doi: 10.1136/eb-2016-102574, PMID: 28073810 PMC10688422

[ref37] Kraepelien M. Schibbye R. Månsson K. Sundström C. Riggare S. Andersson G. . (2020). Individually tailored internet-based cognitive-behavioral therapy for daily functioning in patients with Parkinson’s disease: a randomized controlled trial. J. Parkinsons Dis. 10, 653–664. doi: 10.3233/jpd-191894, PMID: 32176657 PMC7242852

[ref38] Li X. Chen S. Wu S. (2023). Effects of repetitive transcranial magnetic stimulation on sleep and plasma orexin a content in patients with mid-to-late Parkinson’s disease. Chin. J. Phys. Med. Rehabil. 45, 232–235. doi: 10.3760/cma.j.issn.0254-1424.2023.03.008

[ref39] Li L. Tian Z. Zhang X. (2021). Clinical study on electroacupuncture combined with Madopar for sleep disorders in Parkinson disease. J. New Chin. Med. 53, 113–116. doi: 10.13457/j.cnki.jncm.2021.01.030

[ref40] Liu C.-F. Wang T. Zhan S.-Q. Geng D. Q. Wang J. Liu J. . (2018). Management recommendations on sleep disturbance of patients with Parkinson’s disease. Chin. Med. J. 131, 2976–2985. doi: 10.4103/0366-6999.247210, PMID: 30539911 PMC6302643

[ref41] Louter M. van Sloun R. J. G. Pevernagie D. A. A. Arends J. B. A. M. Cluitmans P. J. Bloem B. R. . (2013). Subjectively impaired bed mobility in Parkinson disease affects sleep efficiency. Sleep Med. 14, 668–674. doi: 10.1016/j.sleep.2013.03.010, PMID: 23643658

[ref42] Luo F. Ye M. Lv T. Hu B. Chen J. Yan J. . (2021). Efficacy of cognitive behavioral therapy on mood disorders, sleep, fatigue, and quality of life in Parkinson’s diseases: a systematic review and Meta-analysis. Front. Psychiatry 12:793804. doi: 10.3389/fpsyt.2021.793804, PMID: 34966313 PMC8710613

[ref43] Modugno N. Iaconelli S. Fiorlli M. Lena F. Kusch I. Mirabella G. (2010). Active theater as a complementary therapy for Parkinson’s disease rehabilitation: a pilot study. Sci. World J. 10, 2301–2313. doi: 10.1100/tsw.2010.221, PMID: 21103799 PMC5763766

[ref44] Moon S. Sarmento C. V. M. Steinbacher M. Smirnova I. V. Colgrove Y. Lai S. M. . (2020). Can qigong improve non-motor symptoms in people with Parkinson’s disease—a pilot randomized controlled trial? Complement. Ther. Clin. Pract. 39, 101–169. doi: 10.1016/j.ctcp.2020.101169, PMID: 32379638 PMC7607921

[ref45] Nascimento C. M. C. Ayan C. Cancela J. M. Gobbi L. T. Gobbi S. Stella F. (2013). Effect of a multimodal exercise program on sleep disturbances and instrumental activities of daily living performance on Parkinson’s and Alzheimer’s disease patients. Geriatr. Gerontol. Int. 14, 259–266. doi: 10.1111/ggi.12082, PMID: 23647635

[ref46] Nazarova L. Liu H. Xie H. Wang L. Ding H. An H. . (2022). Targeting gut-brain axis through scalp-abdominal electroacupuncture in Parkinson’s disease. Brain Res. 1790:147956. doi: 10.1016/j.brainres.2022.147956, PMID: 35660372

[ref9001] Nerbass F. B. Feltrim M. I. Z. Souza S. A. de Ykeda D. S. Lorenzi-Filho G. (2010). Effects of massage therapy on sleep quality after coronary artery bypass graft surgery. Clinics (São Paulo, Brazil) 65, 1105–10. doi: 10.1590/s1807-59322010001100008 21243280 PMC2999703

[ref47] Patel S. Ojo O. Genc G. Oravivattanakul S. Huo Y. Rasameesoraj T. . (2017). A computerized cognitive behavioral therapy randomized, controlled, pilot trial for insomnia in Parkinson disease (ACCORD-PD). J. Clin. Mov. Disord. 4:16. doi: 10.1186/s40734-017-0062-2, PMID: 28852567 PMC5568719

[ref48] Paus S. Schmitz-Hübsch T. Wüllner U. Vogel A. Klockgether T. Abele M. (2007). Bright light therapy in Parkinson’s disease: a pilot study. Mov. Disord. 22, 1495–1498. doi: 10.1002/mds.21542, PMID: 17516492

[ref49] Pruthi S. Degnim A. C. Bauer B. A. DePompolo R. W. Nayar V. (2009). Value of massage therapy for patients in a breast clinic. Clin. J. Oncol. Nurs. 13, 422–425. doi: 10.1188/09.cjon.422-425, PMID: 19648098

[ref50] Riemann D. (2019). Sleep, insomnia and neurological and mental disorders. J. Sleep Res. 28:e12892. doi: 10.1111/jsr.12892 31297929

[ref51] Rios Romenets S. Creti L. Fichten C. Bailes S. Libman E. Pelletier A. . (2013). Doxepin and cognitive behavioral therapy for insomnia in patients with Parkinson’s disease—a randomized study. Parkinsonism Relat. Disord. 19, 670–675. doi: 10.1016/j.parkreldis.2013.03.003, PMID: 23561946

[ref52] Rothman S. M. Mattson M. P. (2012). Sleep disturbances in Alzheimer’s and Parkinson’s diseases. Neuromolecular Med. 14, 194–204. doi: 10.1007/s12017-012-8181-2, PMID: 22552887 PMC4544709

[ref53] Rutten S. Vriend C. Smit J. H. Berendse H. W. van Someren E. J. W. Hoogendoorn A. W. . (2019). Bright light therapy for depression in Parkinson disease. Neurology 92, e1145–e1156. doi: 10.1212/wnl.0000000000007090, PMID: 30770426

[ref54] Sacheli M. A. Neva J. L. Lakhani B. Murray D. K. Vafai N. Shahinfard E. . (2019). Exercise increases caudate dopamine release and ventral striatal activation in Parkinson’s disease. Mov. Disord. 34, 1891–1900. doi: 10.1002/mds.27865, PMID: 31584222

[ref55] Salanti G. Del Giovane C. Chaimani A. Caldwell D. M. Higgins J. P. (2014). Evaluating the quality of evidence from a network meta-analysis. PLoS One 9:e99682. doi: 10.1371/journal.pone.0099682, PMID: 24992266 PMC4084629

[ref56] Schootemeijer S. van der Kolk N. M. Bloem B. R. de Vries N. M. (2020). Current perspectives on aerobic exercise in people with Parkinson’s disease. Neurotherapeutics 17, 1418–1433. doi: 10.1007/s13311-020-00904-8, PMID: 32808252 PMC7851311

[ref57] Schroeck J. L. Ford J. Conway E. L. Kurtzhalts K. E. Gee M. E. Vollmer K. A. . (2016). Review of safety and efficacy of sleep medicines in older adults. Clin. Ther. 38, 2340–2372. doi: 10.1016/j.clinthera.2016.09.010 27751669

[ref58] Shan J. Wang Y. Lai C. (2022). Clinical efficacy of high frequency rTMS combined with selegilan hydrochloride in the treatment of PD with excessive daytime sleepiness sleep disorder and its correlation with DTI. Journal of Rehabilitation 37, 592–597.

[ref59] Shi J. Luo D. Weng H. Zeng X. Zeng X. T. Lin L. . (2020). Optimally estimating the sample standard deviation from the five-number summary. Res. Synth. Methods 11, 641–654. doi: 10.1002/jrsm.1429 32562361

[ref60] Silveira H. Moraes H. Oliveira N. Coutinho E. S. F. Laks J. Deslandes A. (2013). Physical exercise and clinically depressed patients: a systematic review and meta-analysis. Neuropsychobiology 67, 61–68. doi: 10.1159/000345160 23295766

[ref61] Skogar Ö. Borg A. Larsson B. Robertsson L. Andersson L. Andersson L. . (2013). ‘Effects of tactile touch on pain, sleep and health related quality of life in Parkinson’s disease with chronic pain’: a randomized, controlled and prospective study. Eur. J. Integr. Med. 5, 141–152. doi: 10.1016/j.eujim.2012.10.005

[ref62] Song P. Lin H. Li S. Wang L. Liu J. Li N. . (2019). Repetitive transcranial magnetic stimulation (rTMS) modulates time-varying electroencephalography (EEG) network in primary insomnia patients: a TMS-EEG study. Sleep Med. 56, 157–163. doi: 10.1016/j.sleep.2019.01.007, PMID: 30871961

[ref63] Stack E. L. Ashburn A. M. (2006). Impaired bed mobility and disordered sleep in Parkinson’s disease. Mov. Disord. 21, 1340–1342. doi: 10.1002/mds.20944, PMID: 16773640

[ref64] Stefani A. Högl B. (2019). Sleep in Parkinson’s disease. Neuropsychopharmacology 45, 121–128. doi: 10.1038/s41386-019-0448-y, PMID: 31234200 PMC6879568

[ref65] Suzuki T. Shimada H. Makizako H. Doi T. Yoshida D. Tsutsumimoto K. . (2012). Effects of multicomponent exercise on cognitive function in older adults with amnestic mild cognitive impairment: a randomized controlled trial. BMC Neurol. 12, 128–135. doi: 10.1186/1471-2377-12-128, PMID: 23113898 PMC3534485

[ref67] Videnovic A. Klerman E. B. Wang W. Marconi A. Kuhta T. Zee P. C. (2017). Timed light therapy for sleep and daytime sleepiness associated with Parkinson disease. JAMA Neurol. 74, 411–418. doi: 10.1001/jamaneurol.2016.5192, PMID: 28241159 PMC5470356

[ref68] Vogel O. Niederer D. Vogt L. (2021). Multimodal exercise effects in older adults depend on sleep, movement biography, and habitual physical activity: a randomized controlled trial. Front. Aging Neurosci. 13, 722–799. doi: 10.3389/fnagi.2021.722799, PMID: 34744686 PMC8570408

[ref69] Wailke S. Herzog J. Witt K. Deuschl G. Volkmann J. (2010). Effect of controlled-release levodopa on the microstructure of sleep in Parkinson’s disease. Eur. J. Neurol. 18, 590–596. doi: 10.1111/j.1468-1331.2010.03213.x, PMID: 20849470

[ref70] Waller R. Gilbody S. (2008). Barriers to the uptake of computerized cognitive behavioral therapy: a systematic review of the quantitative and qualitative evidence. Psychol. Med. 39, 705–712. doi: 10.1017/s0033291708004224, PMID: 18812006

[ref71] Wang C. (2022). Influence of comprehensive music therapy in occupational therapy on emotional cognition and sleep in patients with Parkinson’s disease. RARM 3, 176–178.

[ref72] Wang F. Sun L. Zhang X. Jia J. Liu Z. Huang X. Y. . (2015). Effect and potential mechanism of electroacupuncture add-on treatment in patients with Parkinson’s disease. Evid. Based Complement. Alternat. Med. 2015, 1–11. doi: 10.1155/2015/692795, PMID: 26351515 PMC4550783

[ref73] Wu P. Lee M. Wu S. Ho H. H. Chang M. H. Lin H. S. . (2021). Effects of home-based exercise on motor, non-motor symptoms and health-related quality of life in Parkinson’s disease patients: a randomized controlled trial. Jpn. J. Nurs. Sci. 19:e12418. doi: 10.1111/jjns.12418, PMID: 33876562

[ref74] Wu S. Li X. Qi Y. (2020). The influence of transcranial stimulation on rapid eye movement sleep disorders among persons with Parkinson’s disease. Chin. J. Phys. Med. Rehabil. 42, 50–54. doi: 10.3760/cma.j.issn.0254-1424.2020.01.012

[ref75] Xiao C.-M. Zhuang Y.-C. (2015). Effect of health Baduanjin qigong for mild to moderate Parkinson’s disease. Geriatr. Gerontol. Int. 16, 911–919. doi: 10.1111/ggi.12571, PMID: 26310941

[ref76] Xu Y. Cai X. Qu S. Zhang J. Zhang Z. Yao Z. . (2020). Madopar combined with acupuncture improves motor and non-motor symptoms in Parkinson’s disease patients: a multicenter randomized controlled trial. Eur. J. Integr. Med. 34:101049. doi: 10.1016/j.eujim.2019.101049

[ref77] Yang C. Guo Z. Peng H. Xing G. Chen H. McClure M. A. . (2018). Repetitive transcranial magnetic stimulation therapy for motor recovery in Parkinson’s disease: a meta-analysis. Brain Behav. 8:e01132. doi: 10.1002/brb3.1132, PMID: 30264518 PMC6236247

[ref78] Yong M.-H. Fook-Chong S. Pavanni R. Lim L. L. Tan E. K. (2011). Case control polysomnographic studies of sleep disorders in Parkinson’s disease. PLoS One 6:e22511. doi: 10.1371/journal.pone.0022511, PMID: 21799880 PMC3142152

[ref79] Yu X. Chen H. (2022). Clinical effect and prognosis analysis of repeated transcranial magnetic stimulation in the treatment of patients with Parkinson’s disease and insomnia. China Foreign Med. Treat. 41, 20–24. doi: 10.16662/j.cnki.1674-0742.2022.04.020

[ref80] Zhang H. Gu Z. An J. Wang C. Chan P. (2014). Non-motor symptoms in treated and untreated Chinese patients with early Parkinson’s disease. Tohoku J. Exp. Med. 232, 129–136. doi: 10.1620/tjem.232.129, PMID: 24573063

[ref81] Zhang W. Nie K. Zhang Y. (2014). Observation on the effect of repetitive transcranial magnetic stimulation in the treatment of insomnia in patients with Parkinson’s disease. J. Nurs. 21, 28–30. doi: 10.16460/j.issn1008-9969.2014.23.012

[ref82] Zhang X. Zhuang S. Wu J. Wang L. Mao C. Chen J. . (2022). Effects of repetitive transcranial magnetic stimulation over right dorsolateral prefrontal cortex on excessive daytime sleepiness in patients with Parkinson’s disease. Sleep Med. 100, 133–138. doi: 10.1016/j.sleep.2022.08.003, PMID: 36049407

[ref83] Zhu M. Zhang Y. Pan J. Fu C. Wang Y. (2020). Effect of simplified tai chi exercise on relieving symptoms of patients with mild to moderate Parkinson’s disease. J. Sports Med. Phys. Fitness 60, 282–288. doi: 10.23736/s0022-4707.19.10104-1, PMID: 31665879

[ref84] Zhuang S. Wang F.-Y. Gu X. Wu J. J. Mao C. J. Gui H. . (2020). Low-frequency repetitive transcranial magnetic stimulation over right dorsolateral prefrontal cortex in Parkinson’s disease. Parkinson’s Dis. 2020, 1–7. doi: 10.1155/2020/7295414, PMID: 33005318 PMC7509565

[ref9002] Zuzuárregui J. R. P. During E. H. (2020). Sleep Issues in Parkinson’s Disease and Their Management. Neurotherapeutics, 17, 1480–1494. doi: 10.1007/s13311-020-00938-y 33029723 PMC7851262

