# Supplementary material for: Efficacy of non-pharmacological interventions for sleep quality in Parkinson’s disease: a systematic review and network meta-analysis
Source: Front Neurosci. 2024 Feb 21;18:1337616. doi: 10.3389/fnins.2024.1337616 (PMC10914945; doi:10.3389/fnins.2024.1337616)
Supplement: Supplementary file 2 [file Table_2.docx]

**Table 2 The detailed search strategy**

| **Search** | **Query** | **Items found** |
| --- | --- | --- |
| **1. Database: PubMed** | | |
| #1 | ("Parkinson Disease"[Mesh]) OR ((((Parkinson's Disease[Title/Abstract]) OR (Parkinson*[Title/Abstract])) OR (Paralysis Agitans[Title/Abstract])) OR (PD[Title/Abstract])) | 292,418 |
| #2 | (((((((((non-pharmacolog*[Title/Abstract]) OR (Intervention[Title/Abstract])) OR (treatment[Title/Abstract])) OR (Training[Title/Abstract])) OR (rehabilitation[Title/Abstract])) OR (exercise[Title/Abstract])) OR (therapy[Title/Abstract])) OR (Bright light therapy[Title/Abstract])) OR (BLT[Title/Abstract])) OR (((((((((((((((((((repetitive transcranial magnetic stimulation[Title/Abstract]) OR (rTMS[Title/Abstract])) OR (deep brain stimulation[Title/Abstract])) OR (DBS[Title/Abstract])) OR (cognitive behavioural therapy[Title/Abstract])) OR (CBT[Title/Abstract])) OR (Mindfulness Meditation[Title/Abstract])) OR (Baduanjin[Title/Abstract])) OR (Qigong[Title/Abstract])) OR (Continuous Positive Airway Pressure[Title/Abstract])) OR (CPAP[Title/Abstract])) OR (Tai Chi[Title/Abstract])) OR (Acupuncture[Title/Abstract])) OR (Massage therapy[Title/Abstract])) OR (Muscle relaxation[Title/Abstract])) OR (Aerobic Exercise[Title/Abstract])) OR (resistance training[Title/Abstract])) OR (Yoga[Title/Abstract])) OR (Dance[Title/Abstract])) | 7,848,570 |
| #3 | ("Ultrasonic Therapy"[Mesh]) OR (((((((((Therapies, Ultrasonic[Title/Abstract]) OR (Ultrasonic Therapies[Title/Abstract])) OR (Therapeutic Ultrasound[Title/Abstract])) OR (Ultrasound, Therapeutic[Title/Abstract])) OR (Therapy, Ultrasonic[Title/Abstract])) OR (Ultrasound Therapy[Title/Abstract])) OR (Therapies, Ultrasound[Title/Abstract])) OR (Therapy, Ultrasound[Title/Abstract])) OR (Ultrasound Therapies[Title/Abstract])) | 27,847 |
| #4 | ("Low-Level Light Therapy"[Mesh]) OR ((((((((((((((((((((((((((((((((Light Therapies, Low-Level[Title/Abstract]) OR (Light Therapy, Low-Level[Title/Abstract])) OR (Low Level Light Therapy[Title/Abstract])) OR (Low-Level Light Therapies[Title/Abstract])) OR (Therapies, Low-Level Light[Title/Abstract])) OR (Therapy, Low-Level Light[Title/Abstract])) OR (Photobiomodulation Therapy[Title/Abstract])) OR (Photobiomodulation Therapies[Title/Abstract])) OR (Therapies, Photobiomodulation[Title/Abstract])) OR (Therapy, Photobiomodulation[Title/Abstract])) OR (LLLT[Title/Abstract])) OR (Laser Therapy, Low-Level[Title/Abstract])) OR (Laser Therapies, Low-Level[Title/Abstract])) OR (Laser Therapy, Low Level[Title/Abstract])) OR (Low-Level Laser Therapies[Title/Abstract])) OR (Laser Irradiation, Low-Power[Title/Abstract])) OR (Irradiation, Low-Power Laser[Title/Abstract])) OR (Laser Irradiation, Low Power[Title/Abstract])) OR (Low-Power Laser Therapy[Title/Abstract])) OR (Low Power Laser Therapy[Title/Abstract])) OR (Laser Therapy, Low-Power[Title/Abstract])) OR (Laser Therapies, Low-Power[Title/Abstract])) OR (Laser Therapy, Low Power[Title/Abstract])) OR (Low-Power Laser Therapies[Title/Abstract])) OR (Low-Level Laser Therapy[Title/Abstract])) OR (Low Level Laser Therapy[Title/Abstract])) OR (Low-Power Laser Irradiation[Title/Abstract])) OR (Low Power Laser Irradiation[Title/Abstract])) OR (Laser Biostimulation[Title/Abstract])) OR (Biostimulation, Laser[Title/Abstract])) OR (Laser Phototherapy[Title/Abstract])) OR (Phototherapy, Laser[Title/Abstract])) | 9,663 |
| #5 | ("Music Therapy"[Mesh]) OR (Therapy, Music[Title/Abstract]) | 4,465 |
| #6 | #2 OR #3 OR #4 OR #5 | 7,863,206 |
| #7 | ("Dyssomnias"[Mesh]) OR (((((sleep disorders[Title/Abstract]) OR (Sleep[Title/Abstract])) OR (sleepiness[Title/Abstract])) OR (sleep quality[Title/Abstract])) OR (Insomnia[Title/Abstract])) | 248,638 |
| #8 | ("Randomized Controlled Trial" [Publication Type] OR "Randomized Controlled Trials as Topic"[Mesh] OR "Controlled Clinical Trial" [Publication Type]) OR ((Randomized[Title/Abstract]) OR (Placebo[Title/Abstract])) | 1,187,655 |
| #5 | #1 AND #6 AND #7 AND #8 | 407 |
| **2. Database: EMBASE** | | |
| #1 | 'parkinson disease'/exp OR parkinson* OR 'paralysis agitans':ti,ab,kw OR pd:ti,ab,kw | 508,028 |
| #2 | 'non pharmacolog*' OR intervention:ti,ab,kw OR treatment:ti,ab,kw OR training:ti,ab,kw OR rehabilitation:ti,ab,kw OR exercise:ti,ab,kw OR therapy:ti,ab,kw OR 'bright light therapy':ti,ab,kw OR blt:ti,ab,kw OR 'repetitive transcranial magnetic stimulation':ti,ab,kw OR rtms:ti,ab,kw OR 'deep brain stimulation':ti,ab,kw OR dbs:ti,ab,kw OR 'cognitive behavioural therapy':ti,ab,kw OR cbt:ti,ab,kw OR 'mindfulness meditation':ti,ab,kw OR baduanjin:ti,ab,kw OR qigong:ti,ab,kw OR 'continuous positive airway pressure':ti,ab,kw OR cpap:ti,ab,kw OR 'tai chi':ti,ab,kw OR acupuncture:ti,ab,kw OR 'massage therapy':ti,ab,kw OR 'muscle relaxation':ti,ab,kw OR 'aerobic exercise':ti,ab,kw OR 'resistance training':ti,ab,kw OR yoga:ti,ab,kw OR dance:ti,ab,kw | 10,929,310 |
| #3 | 'music therapy'/exp OR 'therapy, music':ti,ab,kw | 9,256 |
| #4 | 'ultrasound therapy'/exp OR 'ultrasound therapy' OR 'therapies, ultrasonic' OR (therapies, AND ('ultrasonic' OR 'ultrasonic'/exp OR ultrasonic)) OR 'ultrasonic therapies':ti,ab,kw OR 'therapeutic ultrasound':ti,ab,kw OR 'ultrasound, therapeutic':ti,ab,kw OR 'therapy, ultrasonic':ti,ab,kw OR 'ultrasound therapy':ti,ab,kw OR 'therapies, ultrasound':ti,ab,kw OR 'therapy, ultrasound':ti,ab,kw OR 'ultrasound therapies':ti,ab,kw | 32,213 |
| #5 | 'low level laser therapy'/exp OR 'low level laser therapy' OR 'light therapies, low-level' OR (('light' OR 'light'/exp OR light) AND therapies, AND 'low level') OR 'light therapy, low-level':ti,ab,kw OR 'low level light therapy':ti,ab,kw OR 'low-level light therapies':ti,ab,kw OR 'therapies, low-level light':ti,ab,kw OR 'therapy, low-level light':ti,ab,kw OR 'photobiomodulation therapy':ti,ab,kw OR 'photobiomodulation therapies':ti,ab,kw OR 'therapies, photobiomodulation':ti,ab,kw OR 'therapy, photobiomodulation':ti,ab,kw OR lllt:ti,ab,kw OR 'laser therapy, low-level':ti,ab,kw OR 'laser therapies, low-level':ti,ab,kw OR 'laser therapy, low level':ti,ab,kw OR 'low-level laser therapies':ti,ab,kw OR 'irradiation, low-power laser':ti,ab,kw OR 'laser irradiation, low power':ti,ab,kw OR 'low-power laser therapy':ti,ab,kw OR 'low power laser therapy':ti,ab,kw OR 'laser therapy, low-power':ti,ab,kw OR 'laser therapies, low-power':ti,ab,kw OR 'laser therapy, low power':ti,ab,kw OR 'low-power laser therapies':ti,ab,kw OR 'low-level laser therapy':ti,ab,kw OR 'low level laser therapy':ti,ab,kw OR 'low-power laser irradiation':ti,ab,kw OR 'low power laser irradiation':ti,ab,kw OR 'laser biostimulation':ti,ab,kw OR 'biostimulation, laser':ti,ab,kw OR 'laser phototherapy':ti,ab,kw OR 'phototherapy, laser':ti,ab,kw | 28,901 |
| #6 | #2 OR #3 OR #4 OR #5 | 10,947,755 |
| #7 | 'dyssomnia'/exp OR 'sleep disorders'/exp OR sleep:ti,ab,kw OR sleepiness:ti,ab,kw OR 'sleep quality':ti,ab,kw OR insomnia:ti,ab,kw | 507,728 |
| #8 | 'randomized controlled trial'/exp OR 'randomized controlled trial (topic)'/exp OR 'controlled clinical trial'/exp OR 'placebo'/exp OR placebo OR randomized:ti,ab,kw | 1,853,715 |
| #9 | #1 AND #6 AND #7 AND #8 | 3,029 |
| **3. Database: CENTRAL** | | |
| #1 | [Parkinson Disease] explode all trees OR (Parkinson's Disease):ti,ab,kw OR (Parkinson*):ti,ab,kw OR (Paralysis Agitans):ti,ab,kw OR (PD):ti,ab,kw | 49,471 |
| #2 | (non-pharmacolog*):ti,ab,kw OR (Intervention):ti,ab,kw OR (treatment):ti,ab,kw OR (Training):ti,ab,kw OR (rehabilitation):ti,ab,kw OR (exercise):ti,ab,kw OR (therapy):ti,ab,kw OR (Bright light therapy):ti,ab,kw OR (BLT):ti,ab,kw OR (repetitive transcranial magnetic stimulation):ti,ab,kw OR (rTMS):ti,ab,kw OR (deep brain stimulation):ti,ab,kw OR (DBS):ti,ab,kw OR (cognitive behavioural therapy):ti,ab,kw OR (CBT):ti,ab,kw OR (rTMS):ti,ab,kw OR (deep brain stimulation):ti,ab,kw OR (DBS):ti,ab,kw OR (cognitive behavioural therapy):ti,ab,kw OR (CBT):ti,ab,kw OR (Tai Chi):ti,ab,kw OR (Acupuncture):ti,ab,kw OR (Massage therapy):ti,ab,kw OR (Muscle relaxation):ti,ab,kw OR (Aerobic Exercise):ti,ab,kw OR (resistance training):ti,ab,kw OR (Yoga):ti,ab,kw OR (Dance):ti,ab,kw | 1,499,251 |
| #3 | [Music Therapy] explode all trees OR (Therapy, Music):ti,ab,kw | 3,836 |
| #4 | [Ultrasonic Therapy] explode all trees OR (Therapies, Ultrasonic):ti,ab,kw OR (Ultrasonic Therapies):ti,ab,kw OR (Therapeutic Ultrasound):ti,ab,kw OR (Ultrasound, Therapeutic):ti,ab,kw OR (Therapy, Ultrasonic):ti,ab,kw OR (Ultrasound Therapy):ti,ab,kw OR (Therapies, Ultrasound):ti,ab,kw OR (Therapy, Ultrasound):ti,ab,kw OR (Ultrasound Therapies):ti,ab,kw | 17,551 |
| #5 | [Low-Level Light Therapy] explode all trees OR (Light Therapies, Low-Level):ti,ab,kw OR (Light Therapy, Low-Level):ti,ab,kw OR (Low Level Light Therapy):ti,ab,kw OR (Low-Level Light Therapies):ti,ab,kw OR (Therapies, Low-Level Light):ti,ab,kw OR (Therapy, Low-Level Light):ti,ab,kw OR (Photobiomodulation Therapy):ti,ab,kw OR (Photobiomodulation Therapies):ti,ab,kw OR (Therapies, Photobiomodulation):ti,ab,kw OR (Therapy, Photobiomodulation):ti,ab,kw OR (LLLT):ti,ab,kw OR (Laser Therapy, Low-Level):ti,ab,kw OR (Laser Therapies, Low-Level):ti,ab,kw OR (Laser Therapy, Low Level):ti,ab,kw OR (Low-Level Laser Therapies):ti,ab,kw OR (Laser Irradiation, Low-Power):ti,ab,kw OR (Irradiation, Low-Power Laser):ti,ab,kw OR (Laser Irradiation, Low Power):ti,ab,kw OR (Low-Power Laser Therapy):ti,ab,kw OR (Low Power Laser Therapy):ti,ab,kw OR (Laser Therapy, Low-Power):ti,ab,kw OR (Laser Therapies, Low-Power):ti,ab,kw OR (Laser Therapy, Low Power):ti,ab,kw OR (Low-Power Laser Therapies):ti,ab,kw OR (Low-Level Laser Therapy):ti,ab,kw OR (Low Level Laser Therapy):ti,ab,kw OR (Low-Power Laser Irradiation):ti,ab,kw OR (Low Power Laser Irradiation):ti,ab,kw OR (Laser Biostimulation):ti,ab,kw OR (Biostimulation, Laser):ti,ab,kw OR (Laser Phototherapy):ti,ab,kw OR (Phototherapy, Laser):ti,ab,kw | 5,253 |
| #6 | #2 OR #3 OR #4 OR #5 | 1,499,666 |
| #7 | [Dyssomnias] explode all trees OR (sleep disorders):ti,ab,kw OR (Sleep):ti,ab,kw OR (sleepiness):ti,ab,kw OR (sleep quality):ti,ab,kw OR (Insomnia):ti,ab,kw | 57367 |
| #8 | [Randomized Controlled Trial] explode all trees OR [Randomized Controlled Trials as Topic] explode all trees OR [Controlled Clinical Trial] explode all trees OR (Randomized):ti,ab,kw OR (Placebo):ti,ab,kw | 1,344,962 |
| #9 | #1 AND #6 AND #7 AND #8 | 1,587 |
| **4. Database: Web of Science** | | |
| #1 | ((((TS=(Parkinson disease )) OR TS=(Parkinson's Disease )) OR TS=(Parkinson* )) OR TS=(Paralysis Agitans )) OR TS=(PD) | 434,841 |
| #2 | (((((((((((((((((((((((((((TS=(non-pharmacolog*)) OR TS=( Intervention )) OR TS=(treatment )) OR TS=(Training )) OR TS=(rehabilitation )) OR TS=(exercise )) OR TS=( therapy )) OR TS=(Bright light therapy )) OR TS=( BLT)) OR TS=( repetitive transcranial magnetic stimulation )) OR TS=(rTMS)) OR TS=( deep brain stimulation )) OR TS=(DBS )) OR TS=(cognitive behavioural therapy )) OR TS=(CBT)) OR TS=(Mindfulness Meditation )) OR TS=(Baduanjin )) OR TS=(Qigong)) OR TS=(Continuous Positive Airway Pressure)) OR TS=(CPAP)) OR TS=( Tai Chi )) OR TS=(Acupuncture)) OR TS=(Massage therapy)) OR TS=( Muscle relaxation)) OR TS=( Aerobic Exercise)) OR TS=( resistance training )) OR TS=(Yoga )) OR TS=(Dance) | 22,931,044 |
| #3 | (TS=(music therapy)) OR TS=(Therapy, Music) | 18,097 |
| #4 | (((((((((TS=(ultrasound therapy)) OR TS=(Therapies, Ultrasonic)) OR TS=(Ultrasonic Therapies)) OR TS=(Therapeutic Ultrasound)) OR TS=(Ultrasound, Therapeutic)) OR TS=(Therapy, Ultrasonic)) OR TS=(Ultrasound Therapy)) OR TS=(Therapies, Ultrasound)) OR TS=(Therapy, Ultrasound)) OR TS=(Ultrasound Therapies) | 216,497 |
| #5 | ((((((((((((((((((((((((((((((((TS=(low-level laser therapy)) OR TS=(Light Therapies, Low-Level)) OR TS=(Light Therapy, Low-Level)) OR TS=(Low Level Light Therapy)) OR TS=(Low-Level Light Therapies)) OR TS=(Therapies, Low-Level Light)) OR TS=(Therapy, Low-Level Light)) OR TS=(Photobiomodulation Therapy)) OR TS=(Photobiomodulation Therapy)) OR TS=(Photobiomodulation Therapy)) OR TS=(Therapy, Photobiomodulation)) OR TS=(LLLT)) OR TS=(Laser Therapy, Low-Level)) OR TS=(Laser Therapy, Low-Level)) OR TS=(Laser Therapy, Low Level)) OR TS=(Low-Level Laser Therapies)) OR TS=(Laser Irradiation, Low-Power)) OR TS=(Irradiation, Low-Power Laser)) OR TS=(Laser Irradiation, Low Power)) OR TS=(Low-Power Laser Therapy)) OR TS=(Low Power Laser Therapy)) OR TS=(Laser Therapy, Low-Power)) OR TS=(Laser Therapies, Low-Power)) OR TS=(Laser Therapy, Low Power)) OR TS=(Low-Power Laser Therapies)) OR TS=(Low-Level Laser Therapy)) OR TS=(Low Level Laser Therapy)) OR TS=(Low-Power Laser Irradiation)) OR TS=(Low Power Laser Irradiation)) OR TS=(Laser Biostimulation)) OR TS=(Biostimulation, Laser)) OR TS=(Laser Phototherapy)) OR TS=(Phototherapy, Laser) | 43,552 |
| #6 | #2 OR #3 OR #4 OR #5 | 22,951,097 |
| #7 | (((((TS=(Dyssomnias )) OR TS=(sleep disorders )) OR TS=(Sleep )) OR TS=(sleepiness )) OR TS=(sleep quality )) OR TS=(Insomnia) |  |
| #8 | ((((TS=(Randomized Controlled Trial)) OR TS=(Randomized Controlled Trials as Topic)) OR TS=(Controlled Clinical Trial)) OR TS=(Randomized)) OR TS=(Placebo) |  |
| #9 | #1 AND #6 AND #7 AND #8 | 1,104 |
| **5. Database: CNKI** | | |
| #1 | 主题：（帕金森病） AND 主题：（睡眠 + 睡眠障碍 + 失眠 + 睡眠质量 + 嗜睡） AND 主题：（随机 + 随机对照 + 试验） | 108 |
| **6. Database: WANFANG** | | |
| #1 | 主题：（帕金森病） AND 主题：（睡眠 or 睡眠障碍 or 失眠 or 睡眠质量 or 嗜睡） AND 主题：（随机 or 随机对照 or 试验） | 749 |
