# Supplementary figures and images for: Efficacy of non-pharmacological interventions for sleep quality in Parkinson’s disease: a systematic review and network meta-analysis
Source: Front Neurosci. 2024 Feb 21;18:1337616. doi: 10.3389/fnins.2024.1337616 (PMC10914945; doi:10.3389/fnins.2024.1337616)

Table 3A The node-splitting analysis


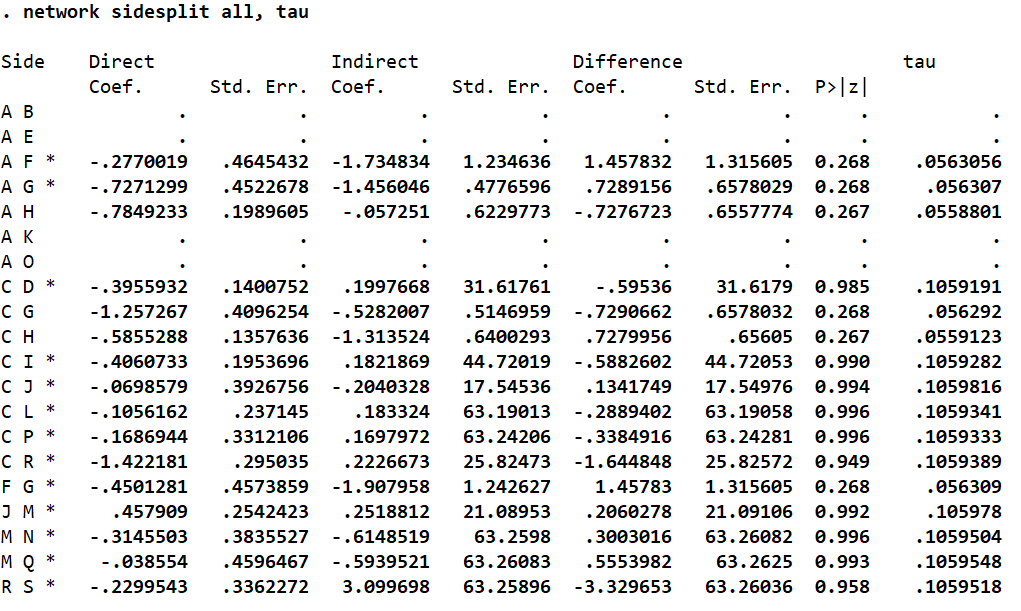


Table 3B Egger's test


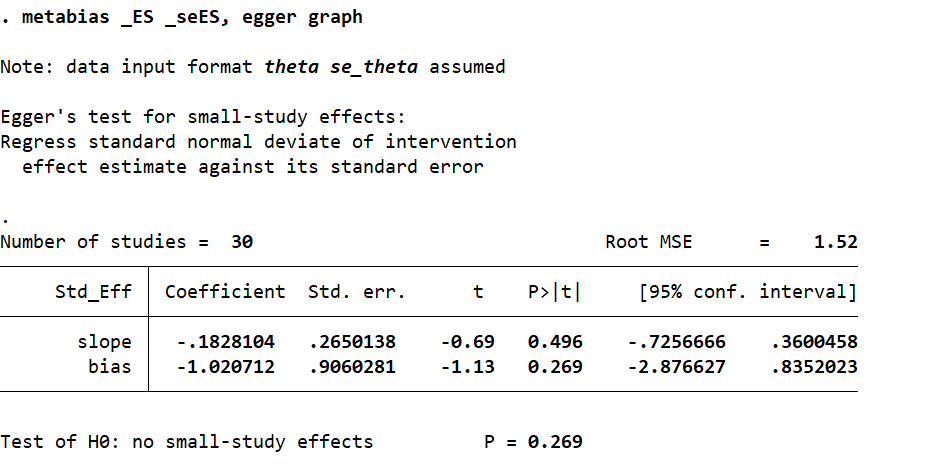

Supplement: Supplementary file 3 [file Table_3.docx]

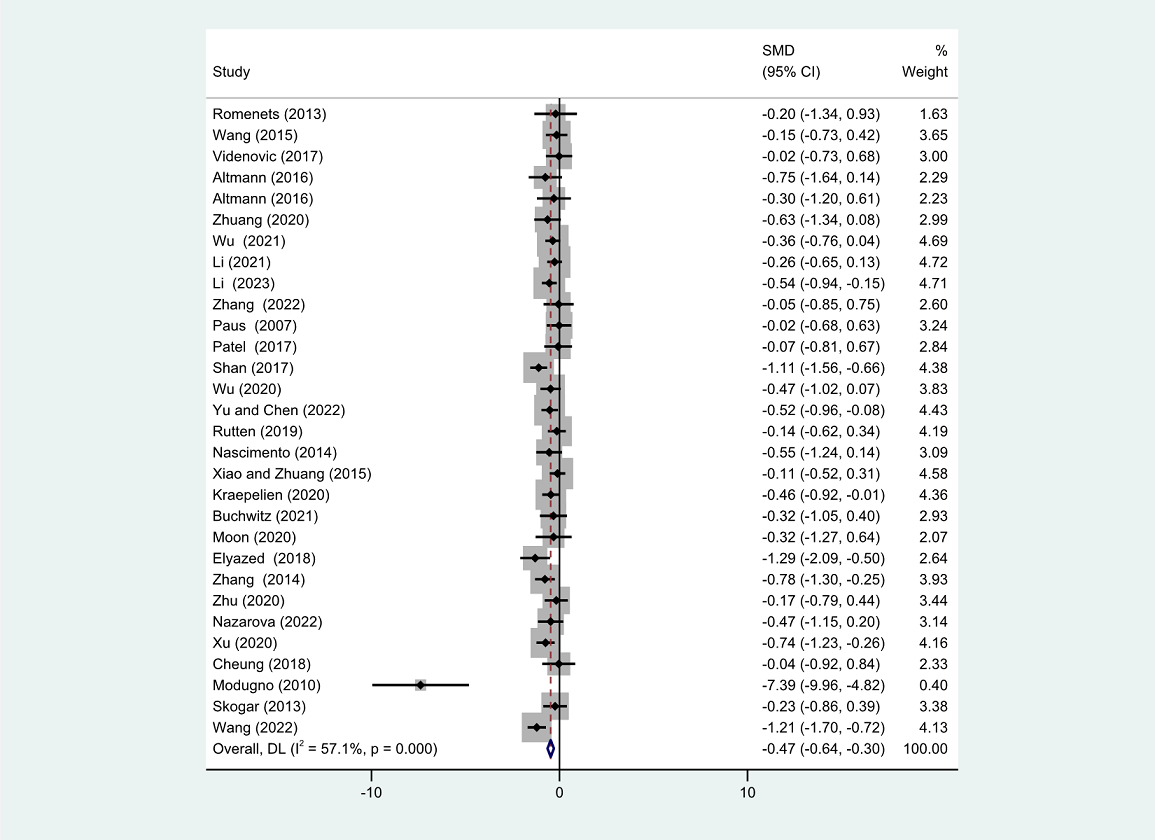

Supplement: Supplementary file 4 [file Image_1.tif]

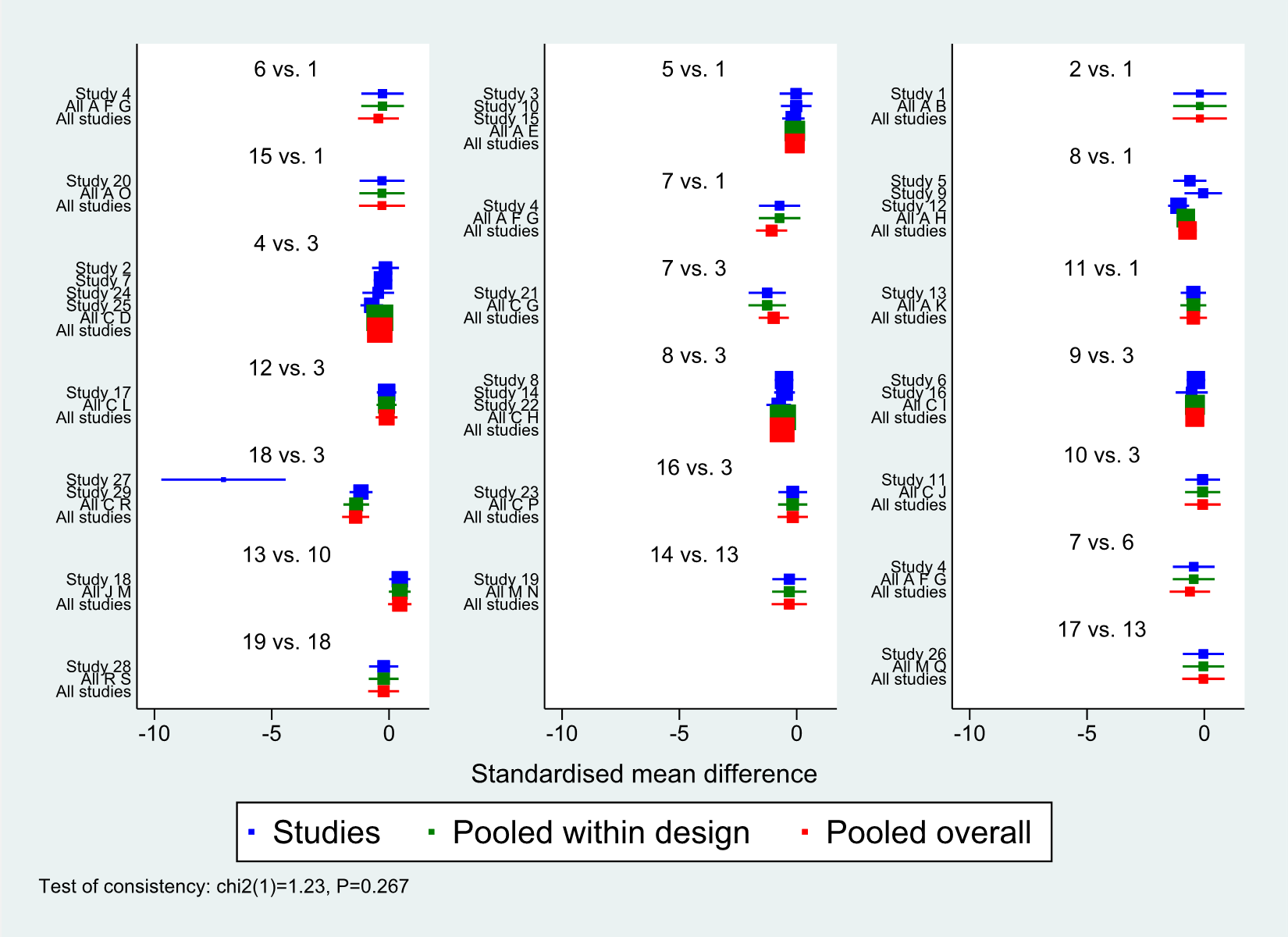

Supplement: Supplementary file 5 [file Image_2.tif]
